# Solubility Characteristics of Acetaminophen and Phenacetin in Binary Mixtures of Aqueous Organic Solvents: Experimental and Deep Machine Learning Screening of Green Dissolution Media

**DOI:** 10.3390/pharmaceutics14122828

**Published:** 2022-12-16

**Authors:** Piotr Cysewski, Tomasz Jeliński, Maciej Przybyłek, Wiktor Nowak, Michał Olczak

**Affiliations:** Department of Physical Chemistry, Pharmacy Faculty, Collegium Medicum of Bydgoszcz, Nicolaus Copernicus University in Toruń, Kurpińskiego 5, 85-096 Bydgoszcz, Poland

**Keywords:** acetaminophen, phenacetin, solubility, screening, machine learning, artificial neural networks, aqueous organic solvents

## Abstract

The solubility of active pharmaceutical ingredients is a mandatory physicochemical characteristic in pharmaceutical practice. However, the number of potential solvents and their mixtures prevents direct measurements of all possible combinations for finding environmentally friendly, operational and cost-effective solubilizers. That is why support from theoretical screening seems to be valuable. Here, a collection of acetaminophen and phenacetin solubility data in neat and binary solvent mixtures was used for the development of a nonlinear deep machine learning model using new intuitive molecular descriptors derived from COSMO-RS computations. The literature dataset was augmented with results of new measurements in aqueous binary mixtures of 4-formylmorpholine, DMSO and DMF. The solubility values back-computed with the developed ensemble of neural networks are in perfect agreement with the experimental data, which enables the extensive screening of many combinations of solvents not studied experimentally within the applicability domain of the trained model. The final predictions were presented not only in the form of the set of optimal hyperparameters but also in a more intuitive way by the set of parameters of the Jouyban–Acree equation often used in the co-solvency domain. This new and effective approach is easily extendible to other systems, enabling the fast and reliable selection of candidates for new solvents and directing the experimental solubility screening of active pharmaceutical ingredients.

## 1. Introduction

Acetaminophen (*N*-(4-hydroxyphenyl)acetamide, CAS: 103-90-2), also known under the name paracetamol, was synthesized as early as 1878 [1] and was later used clinically [2]. Since then, acetaminophen has become one of the most often used and prescribed drugs in the world. Paracetamol mainly acts as an analgesic and antipyretic, has a very weak anti-inflammatory effect and has a low risk of side effects compared to aspirin and nonsteroidal anti-inflammatory drugs [3]. The importance of this drug and its widespread occurrence are the reasons that it is still the subject of many research papers dealing with all aspects of its usage [4,5].

Phenacetin (*N*-(4-ethoxyphenyl) acetamide, CAS: 62-44-2) is structurally similar to acetaminophen but contains an alkoxy group instead of the hydroxyl substituent. It was widely used together with acetaminophen as a pain-relieving and fever-reducing drug until its withdrawal from medicinal usage. Even though phenacetin was, for a long time, used more often than acetaminophen, it was banned by different organizations due to its adverse effects on human health, including carcinogenic and kidney-damaging properties [6,7]. Interestingly, nowadays, it has been found to be one of the main cocaine adulterants worldwide [8,9].

One of the main concerns of the pharmaceutical industry is the utilization of appropriate solvents for all stages of creating a pharmaceutical formulation. This is not a trivial task, since the limited solubility of various active pharmaceutical ingredients (APIs) is widely recognized as a major obstacle [10,11,12]. One of the strategies used to overcome this problem is the selection of a proper solvent system for the studied pharmaceutical formulation, which can be a tedious task. Therefore, a screening stage can be very helpful in order to obtain preliminary data and limit resource-consuming experiments. In this context, the utilization of machine learning can be a very useful approach for solubility predictions and solvent design. The growing awareness in the scientific community about the nonprecedential value of this methodology is associated with expanding the range of free programming ecosystems for direct use, including Scikit-learn [13], TensorFlow [14], PyTorch [15], Keras [16], OpenNN [17] or DeterminedAI [18], to mention only a few representative examples. Bearing this in mind, it is not surprising that many different approaches were used for inferring solubility from different representations of molecular structures. Indeed, recently, Panapitiya et al. [19] assessed the current deep learning architectures for solubility predictions and molecular representations used for depicting patterns between structural molecular properties and measured molecular solubility. Despite great efforts made in the development of deep machine learning models (DMLM) devoted to solubility predictions, there are still serious limitations prohibiting the general use of already developed models [20]. First of all, they concentrate mainly on aqueous solubility [19,21,22,23,24,25,26,27], with few exceptions [21,28,29,30] devoted to studying organic solvents. An additional limitation comes from the fact that the temperature dependence is often not explicitly defined. Moreover, the applicability domain is not always defined, which makes difficult generalizations and predictions for new systems. On the other hand, DMLM requires a huge number of data, which are unavailable for many potentially interesting but sparingly explored solvents or their mixtures. Hence, screening for new and green solvents is limited.

The aim of this paper is threefold. First of all, the theoretical tool for screening effective and environmentally friendly solvents for acetaminophen and phenacetin dissolution was developed. For this purpose, a nonlinear DMLM was developed using a newly developed ensemble of neural networks (ENN). For learning purposes, the curated experimental solubility data taken from the literature and new measurements were used. Molecular descriptors computed within the COSMO-RS [31] framework were used as a molecular representation of the physicochemical properties of the studied systems in saturated solutions. Finally, the applicability of the developed model for screening for new effective, neat and binary solvents mixtures was documented and discussed.

## 2. Materials and Methods

### 2.1. Solubility Data Collection and Curation

**Figure 1:**
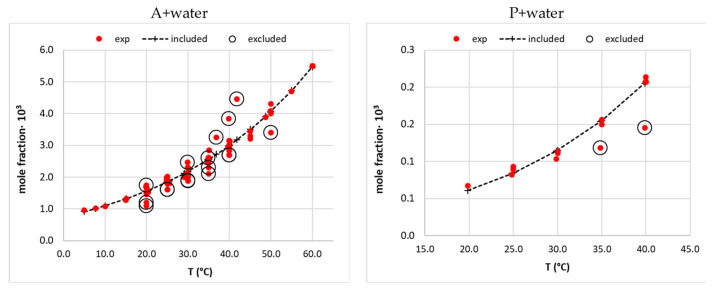
The example of the solubility data curation of acetaminophen (A) [34,35,36,37,38,39,40,41,42,43,44,45,46,47,48] and phenacetin (P) [49,50,51] in neat water. Open circles represent data points with percentage deviations from back-computed values higher than 10% and were excluded from the analysis.

The results of solubility measurements of acetaminophen (A) and phenacetin (P) were searched in the literature. Both active pharmaceutical ingredients were the subjects of quite intensive investigations in an extended range of neat solvents and multicomponent solvent mixtures. Unfortunately, due to differences in the applied methodology, in some cases, quite large deviations could be noticed between the reported values. Since nonlinear models are very sensitive to the quality of the dataset used for training, the observed discrepancies need to be resolved prior to the application of DMLM. Hence, in the very first step, the data have been meticulously analyzed for removing incongruences. This step was done by a careful inspection of the back-computed data obtained after fitting to the Buchowski–Ksiazczak equation [32] and the Jouyban–Acree model [33] in the case of neat and binary solvents, respectively. The former model, also known in the literature as the *λh*-model, is a two-parameter equation that is often used for fitting of the solid–liquid equilibrium data. Formally, the mole fraction of the solute is defined and computed as follows:

(1) xi=λexp(λ·h·(1T−1Tm)−1+λ

where *λ* and *h* are adjustable parameters, and *T_m_* stands for the melting temperature. This equation is very popular and is often used for solubility data interpretation due to its high flexibility and the ability to adequately represent temperature-related solubility. Very often, the accuracy of the back-computed mole fraction is within the experimental uncertainty. The popularity of the *λh*-model is also related to the fact that the parameters have physical meaning, since h represents the energetics of the solubilization, and *λ* is an indicator of the solute association. The curation procedure was performed in a two-step manner. Initial fitting was done using the whole set of data reported for the given solution. Then, outliers were identified by finding cases with a percentage error deviating higher than 10% from the computed values. After removing these points, the final fitting was done, and the obtained parameters of the applied theoretical models were collected in the Supplementary Materials S2 in “neat solvents”. In all cases where data needed to be cured, the ones back-computed with the aid of Equation (1) were used as experimental points. If only single measurements were available, the original experimental data were used. As an illustration of this step, the solubility of A and P in neat water is provided in Figure 1. It is visible that a cloud of points was collected for saturated water solutions of A. The points annotated with open circles are those that were excluded from the pool of data due to not fulfilling the inclusion criterion. The details of all the considered systems are provided in the Supplementary Materials S1 in Tables S1 and S2.

In principle, the Buchowski–Ksiazczak model is also applicable to multicomponent systems, provided that every solvent composition is considered as a new solvent. However, the solubility of A and P in binary solvent mixtures was determined for different compositions, which makes it difficult to fit the data coming from different authors. Thus, another very popular and accurate model was used instead. In the co-solvency literature, the Jouyban–Acree model [33] was proven to be quite accurate. This approach models the excess solubility by fitting with an aid of a polynomial in the following form:

(2) $$ln\left(x_{1}\right)=x_{2}^{*}\cdot ln\left(x_{12}^{sat}\right)+x_{3}^{*}\cdot ln\left(x_{13}^{sat}\right)+\frac{x_{2}^{*}\cdot x_{3}^{*}}{T}\cdot \overset{2}{\underset{i=0}{\sum }}J_{i}\cdot {\left(x_{2}^{*}-x_{3}^{*}\right)}^{i}$$

The three parameters, *J*_0_, *J*_1_ and *J*_2_, are regressed against experimental data. The curation procedure was similarly applied as for neat solvents. Details of the studied systems are provided in the Supplementary Materials S1 in Tables S3 and S4. Additionally, the values of the obtained parameters with statistical measures of accuracy were collected in the Supplementary Materials S2 in “binary solvents”.

### 2.2. Solubility Measurements

#### 2.2.1. Materials

The following analytical-grade compounds were used without any initial procedures. Acetaminophen (A, CAS: 103-90-2) and phenacetin (P, CAS: 62-44-2) were purchased from Sigma-Aldrich (Poznań, Poland), as well as dimethyl sulfoxide (DMSO, CAS: 67-68-5), *N*,*N*-dimethylformamide (DMF, CAS: 68-12-2) and 4-formylmorpholine (4FM, CAS: 4394-85-8). The 5.0-grade nitrogen was obtained from Linde (Warsaw, Poland) and used during the DSC experiments.

#### 2.2.2. Solubility Measurement Protocol

First, an excess amount of either of the APIs was placed in a test tube, which was then filled with 10 mL of solvent, which enabled to obtain saturated solutions. The binary mixtures were prepared by mixing the organic solvent with water in appropriate molar fractions. For each system, three samples were prepared. Samples prepared in such a manner were placed in an Orbital Shaker ES-20/60 incubator from Biosan (Riga, Latvia) and incubated for 24 h at four different temperatures ranging from 20 °C to 40 °C with intervals equal to 5 °C. The temperature was adjusted with ±0.1 °C accuracy, and its daily variance was ±0.5 °C. Shaking of the samples at 60 rev/min was applied together with heating. The next step involved the filtration of the samples using syringes with 0.22 μm pore size PTFE filters. In order to avoid precipitation, the test tubes, syringes and filters were heated before the filtration at appropriate temperatures, matching the ones of the solutions. Next, a small volume of the filtrate was transferred using an automatic pipette to a tube filled with methanol in order to conduct spectroscopic measurements. For the calculation of mole fractions of the solute, the density of the samples was also determined by weighing a fixed amount of the solution in a 10 mL volumetric flask. The calibration curve was prepared with the use of stock solutions of acetaminophen and phenacetin in methanol with concentrations equal to 2.00 · 10^−5^ mg/mL and 1.89 · 10^−5^ mg/mL, respectively. Small amounts of these stock solutions were successfully diluted with methanol in 10 mL volumetric flasks in order to obtain solutions with decreasing concentrations. The absorption of these solutions was measured using an A360 spectrophotometer from AOE Instruments (Shanghai, China). The analytical wavelengths were selected as 249 nm for both acetaminophen and phenacetin. Three separate curves were prepared, and the final curve used for solubility determination was the result of their averaging. The spectra of the prepared samples were measured using the same device with a wavelength range from 215 to 500 nm with a 1 nm resolution. In order to ensure that the absorbance values do not exceed 2.5, the samples were diluted with methanol accordingly. The solubility of acetaminophen and phenacetin in the samples was determined based on the calibration curves, and their mole fractions in the solutions were calculated.

#### 2.2.3. FTIR and DSC Characteristics of Solid Residues

The Fourier-transform infrared spectroscopy (FTIR) and differential scanning calorimetry (DSC) techniques were utilized to analyze the solid residues left after solubility measurements. Before the actual analysis, the samples were left to dry by air. A FTIR Spectrum Two spectrophotometer from Perkin Elmer (Waltham, MA, USA) was used with a diamond attenuated total reflection (ATR) device. For the DSC measurements, the DSC 6000 calorimeter, also from Perkin Elmer (Waltham, MA, USA), was used. The parameters were set as the heating rate of 5 K/min and the nitrogen flow of 20 mL/min. Zinc and indium reference standards were used for calibration, and the samples were placed in standard aluminum pans.

### 2.3. Deep Machine Learning Approach (DML)

Among many available algorithms developed for DML, artificial neural networks (ANNs) are one of the most universal, flexible and effective ways of model development. For the purpose of this project, the simplest form of neural network architecture was implemented, which comprised just one hidden layer in-between the input and output ones. Despite its simplicity, such ANNs can be very effective [28] if an ensemble of neural networks model (ENNM) is constructed. The effectiveness of the network is boosted by tuning the ANN hyperparameters for the most accurate reproduction of the experimental data. Such properties as the number of neurons in the hidden layer, the form of activation functions and the error function were optimized. The training procedure was done after splitting the whole dataset into three subsets, namely training (70%), test (15%) and validation (15%), with the proportions indicated in parentheses. An ensemble of neural networks was constructed by collecting the best ANNs fulfilling the inclusion criteria: (1) accuracy (RMSD < 0.06), (2) precision (number of outliers ≤ 2%) and (3) reliability (predicted solubility within the formal range of log(x) between 0 and 1 for at least 99% of computed values). The applicability domain (AD) [52,53,54] was analyzed for each ANN and the whole ensemble.

### 2.4. Molecular Descriptors

#### 2.4.1. Affinity Descriptors

**Figure 2:**
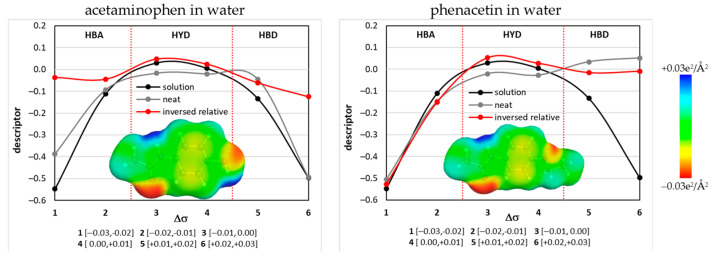
Demonstration of used molecular descriptors defined based on *σ*-potentials.

Despite the very large number of available molecular descriptors, their application to the temperature-related solubility models is not straightforward, and many developed parameters are not intuitive [55]. It seems to be preferable to use such physicochemical properties, which are directly related to the chemical structures in multicomponent systems at defined external conditions. Fortunately, the COMOS-RS framework offers a very intuitive set of chemical system characteristics that might be used for DMLM development. For example [28], the *σ*-chemical potential trends, *µ_s_*(*σ*), are rich in information characterizing the chemical diversity of a given compound in varying environments. It represents the summarized *σ*-profiles with the inclusion of the mixture composition. It is derived [56] by iteratively solving the exact equation:

(3) $$\mu_{s}\left(\sigma \right)=-\frac{RT}{a_{aff}}ln\left[\int^{\;}P_{s}\left(\sigma^{\prime }\right)exp\left\{\frac{a_{aff}}{RT}\left[\mu_{s}\left(\sigma^{\prime }\right)-e\left(\sigma ,\sigma^{\prime }\right)\right]\right\}d\sigma^{\prime }\right]$$

where *σ* represents the charge density; *P_s_*(*σ*) is the so-called *σ*-profile representing the histogram of charge densities; *a_eff_* is an average molecular contact area; $e\left(\sigma ,\sigma^{\prime }\right)$ is the sum of the three (misfit, hydrogen bonding and dispersion) contributions to the intermolecular interaction and RT is the multiplication of the temperature and the gas constant. In Figure 2, there are presented exemplary plots of descriptors derived using *µ_σ_* of A and P in pure water at room temperature. For data reduction purposes, the descriptors are defined as averaged values in the ranges defined in Figure 2 for three meaningful subregions quantifying fundamental affinity properties. It is assumed [56] that the electronegative charge distribution region, *σ* ∈ <−0.03, −0.01>, characterizes the affinity for HB donors, HBA (hydrogen-bonding acceptability). On the opposite scale, there is the electropositive polarity interval *σ* ∈ <0.01, 0.03> to which the affinity for HB acceptors is associated, HBD (hydrogen bond donicity). The intermediate range, *σ* ∈ <−0.01, +0.01>, characterizes contributions to nonpolar interactions and is regarded as a measure of hydrophobicity, HYD. In Figure 2, there are the lines plotted for the pure solute and the solute at an infinite dilution in a given solvent or solvent mixture. Additionally, there are provided plots of relative values of an inverse plot of the solute and solution [28]. These values represent the area between the former plots and were used as molecular descriptors. Hence, the following measures of mutual affinity were used for model training:

1. HBD = HBD_solute_(inversed) − HBA_solvent_—the relative solute donicity with respect to solute acceptability;
2. HBA = HBA_solute_(inversed) − HBD_solvent_—the relative value of solute acceptability with respect to solvent donicity D;
3. HYD = HYD_solute_(inversed) − HYD_solvent_—the relative hydrophobicity measure.

The above definition can be used for deriving the temperature-dependent values of the molecular descriptors for any solute in any solvent or solvent mixture. However, prior to the application of Equation (3), it is necessary to properly represent the molecular structure. This is done after performing a full conformational analysis and identifying the most energetically favorable conformations. For these calculations, COMSOconf software (version 20.0.0, BIOVIA, San Diego, CA, USA) utilized TURBOMOLE (revision V7.5.1, TURBOMOLE GmbH, Karlsruhe, Germany) interfaced with TmoleX 2021 (version 21.0.1, BIOVIA, San Diego, CA, USA). The RI-DFT BP86 (B88-VWN-P86) level of theory was used. The geometry optimization utilized the def-TZVP basis set, while the single point calculations were conducted using the def2-TZVPD basis set. Additionally included were the fine grid tetrahedron cavity and the parameter sets with a hydrogen bond interaction and van der Waals dispersion term based on the “D3” method of Grimme et al. [57]. The final solubility calculations were done with COSMOtherm (version 22.0.0, BIOVIA, San Diego, CA, USA) [58], and the BP_TZVPD_FINE_21.ctd parametrization was used.

#### 2.4.2. Estimated Solubility as a Molecular Descriptor

The COSMO-RS (Conductor-like Screening Model for Real Solvents) [59,60,61,62] is a commonly applied approach for the theoretical characteristics of liquid multicomponent systems. It takes advantage of both quantum chemistry and post-quantum statistical thermodynamics for computing many fundamental properties, including chemical activity. Although this approach cannot deal with solids, the solubility can be still computed provided that fusion properties are known. Fortunately, for both studied aromatic amides, these quantities were measured and reported several times in the literature [63]. For the purpose of solubility computations, the following averaged values were used: *T_m_*(A) = 442.2 K, *T_m_*(P) = 408.1 K, *H_fus_*(A) = 26.90 kJ/mol and *H_fus_*(P) = 30.70 kJ/mol. These values are indispensable, since the activity of the solute in the saturated state and the value of the pure solid phase chemical potential are identical. The latter depends on the fusion properties:

(4) $$ln\left(a_{i}^{s}\right)=ln\left(\gamma_{i}^{sat}x_{i}^{sat}\right)=-\frac{\Delta_{fus}{\bar{G}}_{i}^{m}}{RT}\;$$

where $\Delta_{fus}{\bar{G}}_{i}^{m}$ stands for the partial molar Gibbs free energy of melting at conditions corresponding to the solubility measurements. By definition, this term for the pure solute at its melting point equals zero. The actual solubility computations conducted using COSMO-RS rely on iteratively solving the following equation:

(5) $$ln\left(\gamma_{i}^{sat,i+1}x_{i}^{sat,i+1}\right)=\frac{1}{RT}\left(\mu_{i}^{o,liq}-\mu_{i}^{\left(i\right)}\left(\gamma_{i}^{sat,i}x_{i}^{sat,i}\right)+max\left(0,\Delta_{fus}{\bar{G}}_{i}^{m}\right)\right)$$

Superscripts, *i* and *i* + 1 in the above equation correspond to the values obtained in the two subsequent iterations. The repetition of the iterative cycle lasts until convergence is achieved. The computation completes itself when the computed solubility difference is below a certain threshold value.

**Figure 3:**
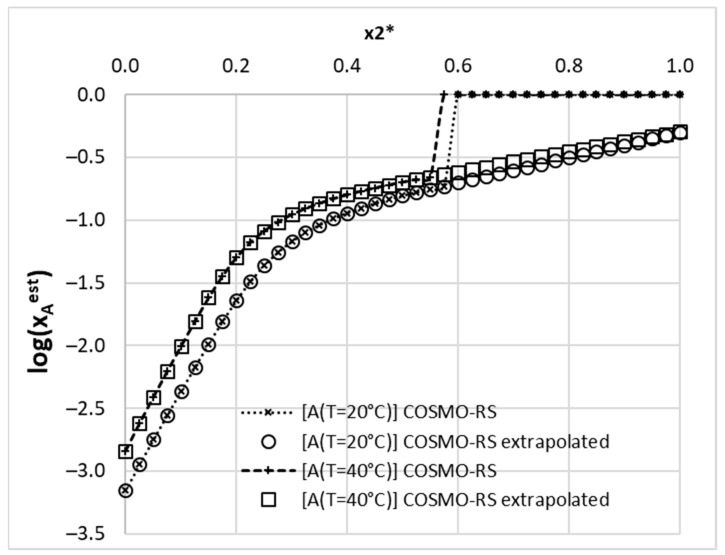
Example of computed solubility extrapolation in the case of the (1) A + (2) DMSO + (3) water system. Lines with crosses correspond to results generated by the COSMOtherm program, and the ones with open symbols were used as a linear interpolation of the last five points.

It is worth admitting that, for many systems already studied, the solubility computations within the COSMO-RS framework are often only qualitatively accurate [64,65,66]. Nevertheless, despite the discrepancies between measured and computed solubility values, they still capture an important part of system information, since the computed values of solubility are quite well correlated with the experimental ones (R^2^ = 0.907). However, in the studied population, the value of the mean average percentage error (PE) exceeds 53% of the mole fraction solubility, and there are cases with PE > 1000%. This means that the accuracy of the computed solubility is not sufficient for screening purposes but can be very effective if used as molecular descriptors for nonlinear modeling. There is also another important issue related to solubility computations using the COSMO-RS approach. For some systems, the complete miscibility of the solute with solvent is obtained. This is illustrated in Figure 3 for acetaminophen. Dotted lines with crosses represent results obtained using COSMOtherm. Unexpectedly, at higher concentrations of DMSO (x_2_* > 0.6), the program fails in solubility computations, indicating complete miscibility. These artificial results were observed also for DMF and 4-formylmorpholine. For the consistent application of computed solubility, extrapolation was used in such cases, as indicated in Figure 3 by plots with open symbols. This prevents from using nonphysical values of solubility as molecular descriptors. Despite these shortcomings, it happened that the selection of the computed solubility as a molecular descriptor is appropriate, since neural networks associate a high contribution to this parameter.

#### 2.4.3. Intermolecular Interactions as Molecular Descriptors

The final set of molecular descriptors comes from the inspection of the solubility files and extraction of the three major contributions to the intermolecular interactions. Within the COSMO-RS framework, bulk systems are modeled as an association of closely packed molecules that are ideally screened and enclosed within a virtual conductor. The Coulomb interactions are the results of the screening of two contacting segments by their surroundings and, in turn, the back-polarization of the solute molecule. This “misfit” results in a specific interaction energy per unit area that represents the electrostatic part of the total energy:

(6) $$E_{MF}\left(\sigma ,\sigma^{\prime }\right)=a_{eff}\frac{a^{\prime }}{2}{\left(\sigma +\sigma^{\prime }\right)}^{2}$$

where *a_eff_* stands for the effective contact area between two surface segments, with α′ being the adjustable parameter. Similarly, hydrogen bonding (H-Bond) can be described by the charge density of the screening of neighboring strongly negative and positive centers for donors and acceptors, respectively. Such interactions are assumed to take place when contact occurs between two sufficiently polar surface pieces with opposite polarity, which is formally defined as:

(7) $$E_{HB}\left(\sigma ,\sigma^{\prime }\right)=a_{eff}c_{HB}min\left(0;min\left(0;\sigma_{don}+\sigma_{HB}\right);min\left(0;\sigma_{acc}-\sigma_{HB}\right)\right)$$

where *σ_HB_* and *σ*′*_HB_* are adjustable parameters. Additionally, the Van der Waals (vdW) interactions occurring between surface segments have to be included in the COSMO-RS model, which can be defined by the following relation:

(8) $$E_{vdW}\left(\sigma ,\sigma^{\prime }\right)=a_{rff}c_{vdW}\left(\sigma ,\sigma^{\prime }\right)=a_{eff}\left(\tau_{vdW}+{\tau^{\prime }}_{vdW}\right)$$

where *τ*_*vdW*_, *τ*_*vdW*′_ and *c*_*vdW*_ are element-specific adjustable parameters. The Van der Waals energy is related only to the type of element present in the atoms experiencing surface contact.

Any prediction made within the COSMO-RS framework results in obtaining these contributions, which makes them directly available from output files of a given computed property. Therefore, such energetic information can be utilized as a set of universal descriptors characterizing the properties of different systems. Here, the values of the total energy, *E_tot_*, of A or P in the given system were used as a molecular descriptor. In addition, the relative values of the contributions to the interaction energies were used. These energetic molecular descriptors were computed for the *i*th components as follows:

(9) $$\Delta E_{j}^{i}=\frac{E_{j}^{i}}{\sum_{j}E_{j}^{i}}$$

where *j* corresponds to misfit, HB or vdW contributions. The descriptor values for multicomponent systems were computed as sums weighted by molar fractions. This set of molecular descriptors, taking advantage of intermolecular interactions, was already demonstrated [67] as adequate for solubility modeling.

### 2.5. Predictive Models Used for Data Curation and the Presentation of the Final Predictions

Although DML models offer very valuable help in predicting physicochemical properties, the direct application of the obtained model is prohibited by the necessity of demonstrating at least some elementary programming skills in Python or another high-level programming language. For overcoming this limitation, the final predictions were provided in a more intuitive way of application in terms of the parameters of the Jouyban–Acree model. For this purpose, the final ENNM was used for solubility data predictions, and the obtained values were fitted with an aid of Equation 2 (see Supplementary Materials S2 in “screening A” and “screening P”).

### 2.6. Statistical Measures

The three following commonly used statistical measures were used in this paper. Firstly, the percentage error, *PE*, with the following general formula:

(10) $$PE=\frac{\left|x^{exp-}x^{est}\right|}{x^{exp}}\cdot 100\%$$

where *x^exp^* is the experimental value, and *x^est^* is the estimated value. Secondly, the mean averaged percentage error, *MAPE*, described as:

(11) $$MAPE=\frac{1}{N}\sum_{i=1}^{N}\left|\frac{\left(x_{i}^{est}-x_{i}^{exp}\right)}{x_{i}^{exp}}\right|\cdot 100\%$$

where *x*_1_*^est^* is the estimated mole fraction value, *x*_1_*^exp^* is the experimental mole fraction value and *N* is the number of experimental points. Finally, the root means squared deviation, *RMSD*, defined as:

(12) $$RMSD=\sqrt[{\;}]{\frac{1}{N}\sum_{i=1}^{N}{\left(x_{i}^{est}-x_{i}^{exp}\right)}^{2}}\;$$

## 3. Results and Discussion

### 3.1. Experimental Solubility Screening

Inspired by previous successes in the solubility screening of such active pharmaceutical ingredients (APIs) as sulfamethizol [28], phenacetin [64], sulfanilamide [68] and benzamide analogs [69], three aqueous binary mixtures of DMSO, DMF and 4FM were used for solubility measurements. Noteworthy, 4FM is considered a green solvent, which can be used instead of DMF [28,70,71,72,73,74]. All temperature-related solubility profiles measured in this study for pure solvents (acetaminophen in DMSO, DMF and 4FM, phenacetin in 4FM), along with the literature data [34,35,36,37,38,39,40,41,42,43,44,45,46,47,48,49,50,51,75,76,77,78,79,80], were presented in the Supplementary Materials S1 in Tables S1 and S2. The solubility profiles determined for the corresponding aqueous binary mixtures are summarized in the Supplementary Materials S1 in Tables S3 and S4. The exact solubility values are tabularized below in Table 1. DSC and FTIR-ATR analyses were performed for the solid residues obtained after the solubility measurements, and the results were compared with pure acetaminophen and phenacetin (see Supplementary Materials S1 in Figures S1 and S2). As it can be inferred, solubility measurements did not induce any polymorphic or pseudo-polymorphic transitions of acetaminophen and phenacetin.

### 3.2. Characteristics of the Dataset

The whole collection of acetaminophen and phenacetin solubility is provided in detail in the Supplementary Materials. The solubility of acetaminophen was measured in twenty neat solvents, including water [34,35,36,37,38,39,40,41,42,43,44,45,46,47,48], methanol [38,79], ethanol [35,37,46], 1-propanol [41,79], 2-propanol [42,43,75,79], 1-butanol [79], 1-octanol [75,77], propylene glycol [35,36,37], transcutol [37], ethyl acetate [46,76,79], isopropyl myristate [51], acetone [43,76,79], acetonitrile [34,79], 1,4-dioxane [45], hexane [77], cyclohexane [51] and chloroform [51], augmented with measurements conducted in this work for DMF, DMSO and 4-formylmorpholine. Saturated systems of phenacetin are available for such neat solvents as water [49,50,51], methanol [49,78,80], ethanol [78,80], 1-propanol [78,80], 2-propanol [78], 1-butanol [78,80], 2-butanol [78], 1-pentanol [80], 1-octanol [77], ethyl formate [78], ethyl acetate [78,80], *n*-propyl acetate [78], isopropyl myristate [51], acetonitrile [49,78], 1,4-dioxane [49,50], DMF [49,78], DMSO [49], *N*,*N*-dimethylacetamide [78], THF [80], hexane [77], cyclohexane [51], benzene [80] and chloroform [51], supplemented by new measurements reported here in 4-formylmorpholine. Additionally, binary solvents were also explored as potential solubilizing media. The collection for acetaminophen encompasses the following systems: methanol + water [38], ethanol + water [35,37,46,48,81], acetaminophen + propanol [41], isopropanol + water [42], propylene glycol + water [35,36,37], transcutol + water [39], acetonitrile + water [34], 1,4-dioxane + water [45], ethanol + propylene glycol [47] and ethanol + ethyl acetate [46], enriched with the data reported here for the 4-formylmorpholine + water, DMF + water and DMSO + water systems. Moreover, the following six binary solvent mixtures were used for solubility measurements of phenacetin, namely methanol + water [49,82], 1,4-dioxane + water [49,50], acetonitrile + water [49] and DMF + water [49], supplemented with the 4-formylmorpholine + water system studied here. Noteworthy, in the case of acetaminophen in aqueous acetonitrile, aqueous 1,4-dioxane, ethanol-propylene glycol and ethanol-ethyl acetate, a characteristic significant co-solvation effect occurred. In the case of phenacetin, this effect can be noted for 1,4-dioxane–water and acetonitrile–water binary solvents.

**Figure 4:**
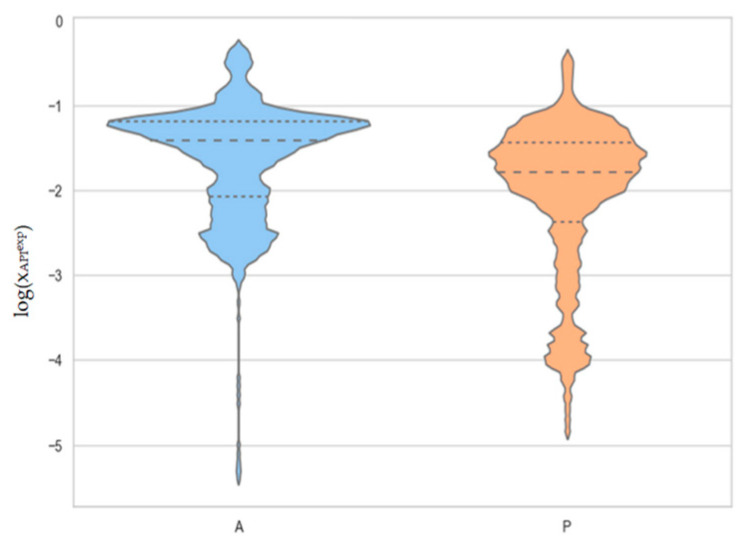
Distribution of the measured solubility values of acetaminophen (A) and phenacetin (P) included in the dataset for model development presented in the form of violin plots. The horizontal lines denote quartiles.

It is necessary to emphasize that this collection, although rich and diverse, is inadequate for the direct use for the training of DMLM. As detailed in the Supplementary Materials, in many cases, the data needed to be cured. The final dataset is synthesized in Figure 4. The diversity of the solubility of acetaminophen and phenacetin is of four orders of magnitude due to the inclusion of highly polar, proto-donating solvents and, also, nonpolar aprotic ones.

**Figure 5:**
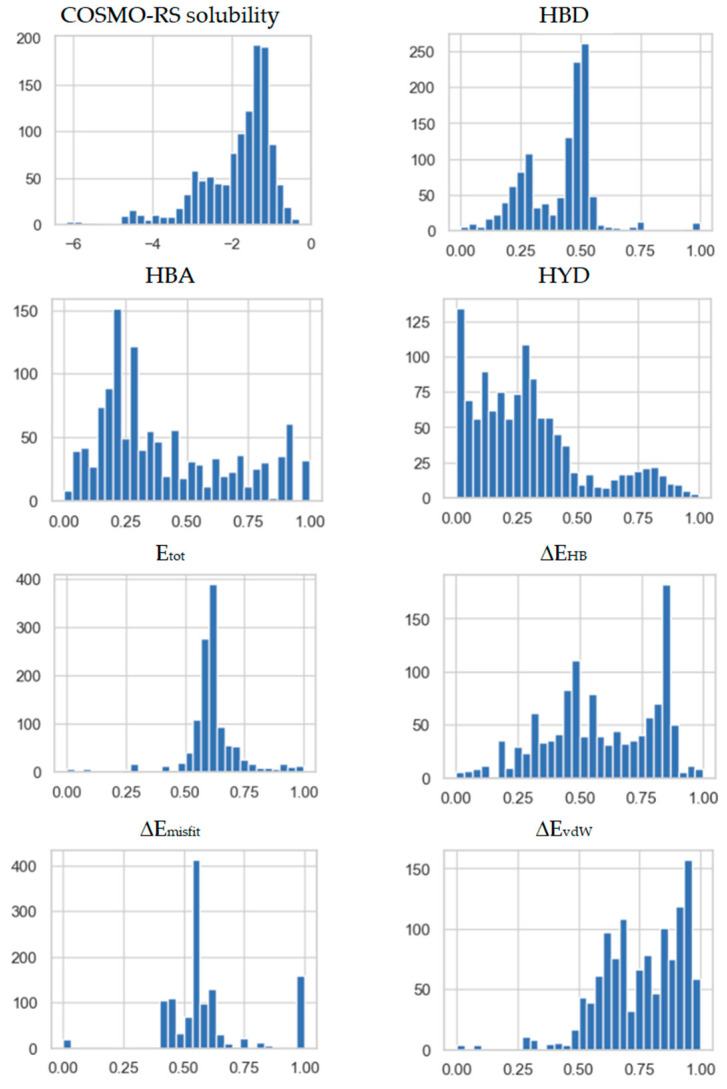
The collection of histograms of normalized values of molecular descriptors used for DMLM development. The following notation is used: HBA and HBD denote hydrogen-bonding affinity and donicity, respectively. HYD denotes hydrophobicity. ΔE_j_ describes relative interaction energies of the solute with respect to the solvent, where *j* = tot stands for the total energy, *j* = HB for the hydrogen bonding, *j* = *vdW* for the dispersion contribution and *j* = misfit for the electrostatic contribution.

The distributions of the molecular descriptors are documented in Figure 5. The three types of molecular descriptors characterize the solute activity in saturated systems expressed by computed solubility, the energetics of intermolecular interactions and charge density distributions interpreted in terms of solute–solvent affinities.

### 3.3. Developed Model of Solubility

**Figure 6:**
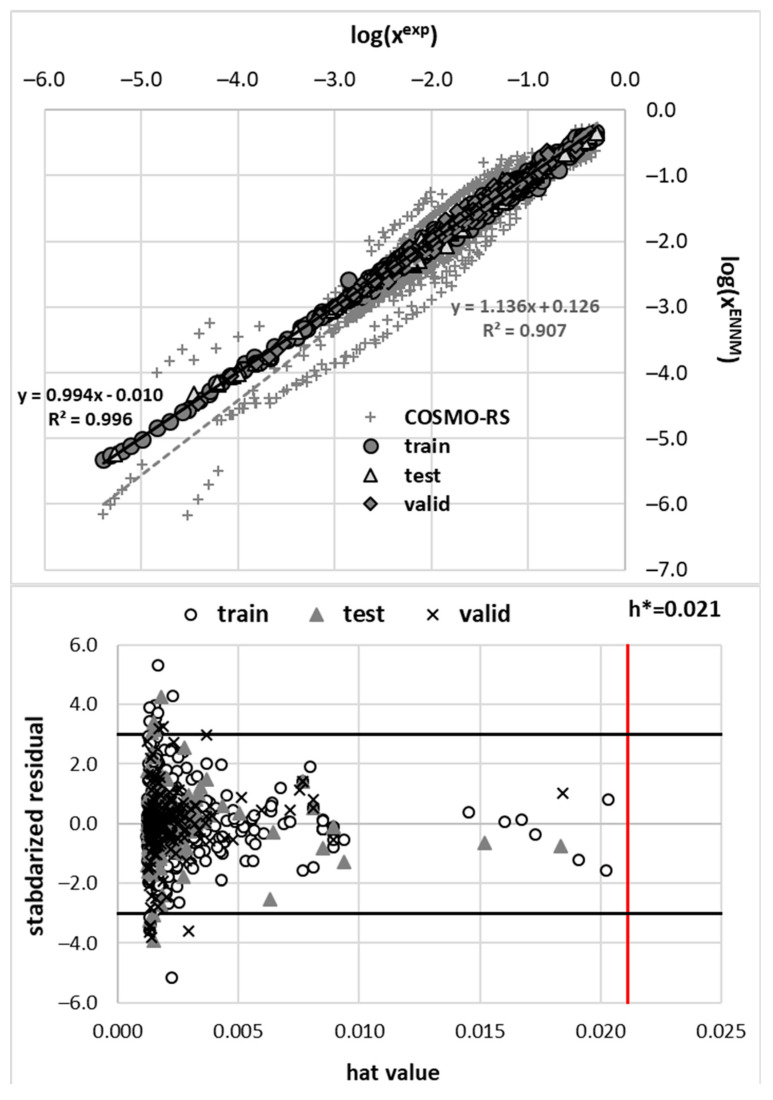
The characteristics of the developed ENMM trained on the A and P 1220 solubility data points using eight molecular descriptors. The top panel characterizes the predictive abilities by splitting them into train, test and validation subsets. Additionally, a comparison between experimental and COSMO-RS-derived solubility is shown. The bottom panel provides an applicability domain analysis done for the final model.

The performed training of the ANNs resulted in an ensemble model, the performance of which is documented in Figure 6. An acceptable accuracy was obtained with R^2^ = 0.996. The number of outliers was less than 2%, defined by three normalized standard deviations.

### 3.4. Solubility Screening

For screening purposes, hypothetical aqueous binary mixtures of green organic solvents were tested. As the measure of environmental friendliness, the values of the indices defined by Harten et al. [83] were used. Solvent substitution is possible using PARIS III (Program for Assisting the Replacement of Industrial Solvents III, Version 1.4.0) software supported by the U.S. Environmental Protection Agency (EPA). PARIS III utilizes a series of computed indices assessing human toxicity potential upon ingestion or inhalation, as well as potentials arising from terrestrial, aquatic, ozone depletion, global warming, photochemical oxidation and acid rain toxicity. In particular, the environmental index (EI) was used as an indicator of the overall relative measure of hazardous properties. For screening purposes, only such solvents were selected that are characterized by EI < 0.5 [83]. This tight criterion unexpectedly excludes DMSO, despite that it is generally considered a green solvent. The reason for this is a very high EI = 11.7 value of DMSO coming from the photochemical oxidation potential contribution. Hence, the selection was done by arbitrarily excluding this contribution, which resulted in the reduction of the EI value of DMSO down to 0.26. It is worth noting that the final list of potential green solvents does not include some commonly used alcohols, starting from methanol and ending on 1-octanol. Additionally, many often-used esters are not included. 4-formylmorpholine is characterized by EI = 0.51, so it can be considered almost green using the criterion adopted for the purpose of this study. The final list of solvents used for screening was shortened from all 5200 available in PARIS III down to 50. The results of the screening are provided in the Supplementary Materials S2 in “screening A” and “screening P”.

**Figure 7:**
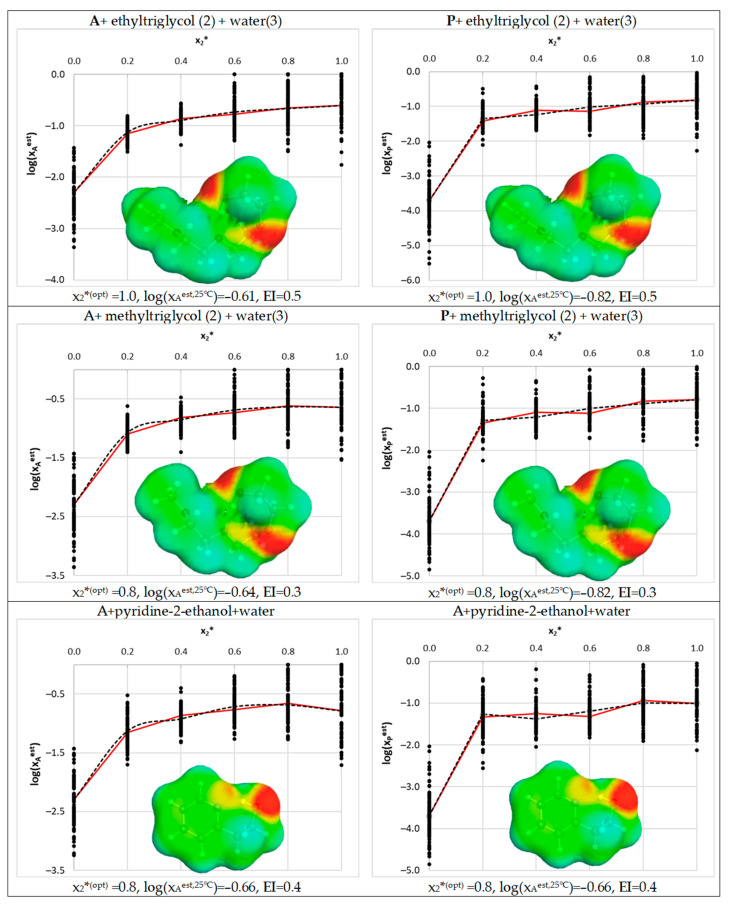
Results of the theoretical green solvents screening inferred from ENNM (T = 25 °C). Electron density scales are the same as in Figure 2. The red line represents solubilities predicted based on parameters fitted with Equation (2), while the dashed one stands for average ENNM solubility data.

The experimentally observed acetaminophen solubility was proven to be the highest in the case of DMF + water (x_2_*^(opt)^ = 0.8, log(x_A_^exp,25°C^) = −0.45, EI = 2.0), followed by DMSO + water (x_2_*^(opt)^ = 0.8, log(x_A_^exp,25°C^) = −0.50, EI = 0.2) > neat 4FM (log(x_A_^exp,25°C^) = −0.78, EI = 0.5), 1,4-dioxane + water (x_2_*^(opt)^ = 0.5, log(x_A_^exp,25°C^) = −0.87, EI = 0.8) > neat transcutol (log(x_A_^exp,25°C^) = −0.95, EI = 0.7). Among these top-best solvents, only DMSO and 4FM can be regarded as green ones, according to the tight criteria set for the purpose of this study. Theoretical screening confirmed the above sequence and additionally enabled the identification of greener alternatives. It happened that the highest solubility of acetaminophen was found for DMSO, which was followed by neat ethyltriglycol and aqueous solutions of methyltriglycol and 2-pyridin-2-ylethanol. The exemplary solubility profiles are collected in Figure 7.

In general, the solubility of phenacetin is slightly lower compared to acetaminophen, and the experimentally observed sequence includes the following neat solvents: *N*,*N*-dimethylacetamide (log(x_P_^exp,25°C^) = −0.67, EI = 1.6), DMF (log(x_P_^exp,25°C^) = −0.93, EI = 2.2) and 4FM (log(x_P_^exp,25°C^) = −1.21, EI = 0.5). Additionally, 1,4-dioxane + water (x_2_*^(opt)^ = 0.65, log(x_A_^exp,25°C^) = −1.33, EI = 0.8) has a relatively high potential of P dissolution. Unfortunately, the solvents that have been used so far are hardly green, with the exception of 4FM. Hence, it is interesting to inspect the results of the theoretical screening. In the case of phenacetin, the screening procedure pointed to aqueous solutions of oleic acid, linoleic acid and octanoic acid as the potentially most-suited solvents. Unfortunately, these solvents are immiscible with water and cannot be used in practice. The aspect of miscibility was not a part of the model development. Probably the replacement of water with DMSO might help in obtaining the homogeneous mixture, which would also result in a green solvent (EI ≈ 0.2). Fortunately, the next solvents found on the prediction list are the same as the ones identified for acetaminophen. Hence, as documented in Figure 7, ethyltriglycol, methyltriglycol and pyridine-2-ethanol are suggested as first-choice solvents for future experiments with acetaminophen and phenacetin.

## 4. Conclusions

The study was aimed at finding green and effective solvents adequate for acetaminophen and phenacetin processing in pharmaceutical practice. This goal was achieved via both the experimental and theoretical screening of binary aqueous solvent mixtures. The latter was achieved by formulating the ensemble neural network model, ENNM, which was derived after representing the molecular features of the studied compounds by using COSMO-RS-derived molecular descriptors. However, the experimental pool of data was cured prior to model development and extended with newly measured data. The considered aqueous binary solutions in which A and P were measured followed the previously observed high solubilizing potential of 4-formylmorpholine, which can also be considered a green solvent (EI = 0.51). This intuition was correct, since acetaminophen is highly soluble in neat 4FM, although the solubility in such solvents as DMF and DMSO is slightly higher. 4-formylmorpholine was also identified as a fairly effective solvent for phenacetin dissolution, although the absolute solubility values are much lower compared to acetaminophen. A closer inspection into the type of solvents used so far for solubility studies of both considered aromatic amides revealed that environmentally friendly solvents were sparingly used. This was the direct motivation for the extensive screening of greener solvents, which are purchasable at a reasonable price. After shortening the list of candidates included in the PARIS III database, comprising 5200 solvents, by applying these criteria, the final screening was done for 50 solvents and their mixtures with water. It was found that both aromatic amides studied here can be effectively dissolved in such green solvents as ethyltriglycol, methyltriglycol and pyridine-2-ethanol. It is very tempting to perform appropriate measurements in the future for confirming these findings. Finally, it is worth adding that the newly developed model for the solubility prediction of acetaminophen and phenacetine relies on three types of criteria for the selection of the neural networks into an ensemble. The first one addresses the model accuracy, which is evaluated using the RMSD measure of every ANN. The second criterion is the model precision, which is meant as a minimization of the number of outliers lying beyond three times the normalized standard deviation. The third criterion addresses reliability, including restrictions on the formal range of the predicted values. In the case of solubility expressed as logarithmic values of mole fractions, this range restricts the predicted values to the span between 0 and 1. Hence, this triple combination of accuracy–precision–reliability criteria is an essential part of the developed ENNM.

## Figures and Tables

**Table 1:** The solubility values (mole fractions) of acetaminophen (A) and phenacetin (P) determined in this study for aqueous–organic binary solvents at different temperatures (x_2_* stands for a mole fraction of the organic component in the binary aqueous–organic solvent).

x_2_*	25 °C	30 °C	35 °C	40 °C
**A + DMSO + water (x_A_ × 10^2^)**				
**0.0**	0.20 ± 0.01	0.24 ± 0.01	0.28 ± 0.01	0.32 ± 0.01
**0.2**	5.17 ± 0.05	7.22 ± 0.38	9.98 ± 0.17	13.66 ± 0.51
**0.4**	13.65 ± 0.37	18.37 ± 0.21	23.79 ± 0.92	31.47 ± 1.15
**0.6**	26.47 ± 0.89	31.69 ± 0.99	37.95 ± 0.67	45.23 ± 0.79
**0.8**	31.61 ± 0.95	37.03 ± 0.96	43.38 ± 0.54	51.08 ± 0.78
**1.0**	30.30 ± 0.55	35.76 ± 0.71	42.22 ± 1.02	48.92 ± 0.62
**A + DMF + water (x_A_ × 10^2^)**				
**0.2**	5.99 ± 0.35	7.65 ± 0.26	9.43 ± 0.40	11.81 ± 0.65
**0.4**	16.13 ± 0.39	19.38 ± 0.46	23.60 ± 1.18	28.88 ± 0.44
**0.6**	31.07 ± 0.28	34.57 ± 1.19	39.04 ± 0.74	44.62 ± 1.60
**0.8**	35.26 ± 0.90	38.58 ± 0.55	43.15 ± 1.19	49.31 ± 1.40
**1.0**	28.69 ± 0.60	31.85 ± 0.56	36.14 ± 0.68	42.73 ± 0.46
**A + 4FM + water (x_A_ × 10^2^)**				
**0.2**	5.18 ± 0.34	7.10 ± 0.38	9.56 ± 0.26	12.54 ± 0.71
**0.4**	9.63 ± 0.19	13.07 ± 0.43	16.68 ± 0.5	20.48 ± 0.47
**0.6**	12.97 ± 0.50	16.89 ± 0.28	21.58 ± 0.82	26.16 ± 0.85
**0.8**	15.41 ± 0.46	19.46 ± 0.55	24.45 ± 0.84	29.54 ± 0.22
**1.0**	16.73 ± 0.55	20.80 ± 0.73	25.89 ± 0.27	31.11 ± 0.73
**P + 4FM + water (x_p_ × 10^2^)**				
**0.2**	0.59 ± 0.03	0.79 ± 0.02	1.08 ± 0.05	1.54 ± 0.04
**0.4**	2.53 ± 0.01	3.02 ± 0.14	3.64 ± 0.19	4.67 ± 0.16
**0.6**	4.19 ± 0.09	4.80 ± 0.01	5.78 ± 0.22	7.11 ± 0.32
**0.8**	5.33 ± 0.07	6.03 ± 0.15	6.94 ± 0.17	8.20 ± 0.07
**1.0**	6.19 ± 0.18	6.89 ± 0.14	7.90 ± 0.13	9.12 ± 0.21

## Data Availability

Not applicable.

## Supplementary Materials

The following supporting information can be downloaded at: https://www.mdpi.com/article/10.3390/pharmaceutics14122828/s1: Supplementary Materials S1: Figure S1. The FTIR-ATR spectra (A) and DSC thermograms (B) measured for acetaminophen solid residues and pure reagent. Figure S2. The FTIR-ATR spectra (A) and DSC thermograms (B) measured for phenacetin solid residues and pure reagent. Table S1. The results of data curation for acetaminophen in neat solvents. Data points were excluded from fitting the *λ h*-equation if PE > 10%. The optimized values of the parameters of the Buchowski–Ksiazczak model applied to acetaminophen solubility in neat solvents are provided in the table in an additional supporting file (see Supplementary Materials S2 in “neat solvents”). Table S2. The results of the data curation for phenacetin in neat solvents. Data points were excluded from fitting the *λ h*-equation if PE > 10%. The optimized values of the parameters of the Buchowski–Ksiazczak model applied to phenacetin solubility in neat solvents are provided in the table in an additional supporting file (see Supplementary Materials S2 in “neat solvents”). Table S3. The data collection of acetaminophen solubility in binary solvent mixtures. The optimized values of the parameters of the Jouyban–Acree model applied to acetaminophen solubility in binary solvents are provided in the table in an additional supporting file (see Supplementary Materials S2 in “binary solvents”). Table S4. The data collection of phenacetin solubility in binary solvent mixtures. The optimized values of the parameters of the Jouyban–Acree model applied to phenacetin solubility in binary solvents are provided in the table in an additional supporting file (see Supplementary Materials S2 in “binary solvents”). Supplementary Materials S2: The obtained model parameters with statistical measures of accuracy for neat and binary solvents, and the screening results for acetaminophen and phenacetin.
