# Supplementary material for: Solubility Characteristics of Acetaminophen and Phenacetin in Binary Mixtures of Aqueous Organic Solvents: Experimental and Deep Machine Learning Screening of Green Dissolution Media
Source: Pharmaceutics. 2022 Dec 16;14(12):2828. doi: 10.3390/pharmaceutics14122828 (PMC9781932; doi:10.3390/pharmaceutics14122828)
Supplement: Supplementary file 1 [file pharmaceutics-14-02828-s001.zip › Supplementary S1.pdf]

# Supplementary Materials for Solubility characteristics of acetaminophen and phenacetin in binary mixtures of aqueous organic solvents: experimental and deep machine learning screening of green dissolution media

Piotr Cysewski *, Tomasz Jeliński*, Maciej Przybyłek, Michał Olczak and Wiktor Nowak

*Department of Physical Chemistry, Pharmacy Faculty, Collegium Medicum of Bydgoszcz, Nicolaus Copernicus University in Toruń, Kurpińskiego 5, 85-096 Bydgoszcz, Poland*

* Correspondence: piotr.cysewski@cm.umk.pl; tomasz.jelinski@cm.umk.pl

## Table of contents

**Figure S1.** The FTIR-ATR spectra (A) and DSC thermograms (B) measured for acetaminophen solid residues and pure reagent. ........ 2

**Figure S2.** The FTIR-ATR spectra (A) and DSC thermograms (B) measured for phenacetin solid residues and pure reagent. ........ 3

**Table S1.** The results of data curation for acetaminophen in neat solvents [1,2,11–20,3–10]. Data points were excluded from fitting λh-equation if PE>10%. The optimized values of parameters of the Buchowski-Ksiazczak [21] model applied to acetaminophen solubility in neat solvents are provided in the table in an additional supporting file (see Supplementary Materials S2 in "neat solvents"). ........ 4

**Table S2.** The results of data curation for phenacetin in neat solvents [19,22–25]. Data points were excluded from fitting λh-equation if PE>10%. The optimized values of parameters of the Buchowski-Ksiazczak [21] model applied to phenacetin solubility in neat solvents are provided in the table in an additional supporting file (see Supplementary Materials S2 in"neat solvents"). ........ 9

**Table S3.** The data collection of acetaminophen solubility in binary solvent mixtures [1,2,13,14,26,3,4,7–12]. The optimized values of parameters of the Jouyban-Acree model [27] model applied to acetaminophen solubility in binary solvents are provided in the table in an additional supporting file (see Supplementary Materials S2 in "binary solvents"). ........ 15

**Table S4.** The data collection of phenacetin solubility in binary solvent mixtures [22,23,28]. The optimized values of parameters of the Jouyban-Acree model [27] model applied to phenacetin solubility in binary solvents are provided in the table in an additional supporting file (see Supplementary Materials S2 in "binary solvents"). ........ 19

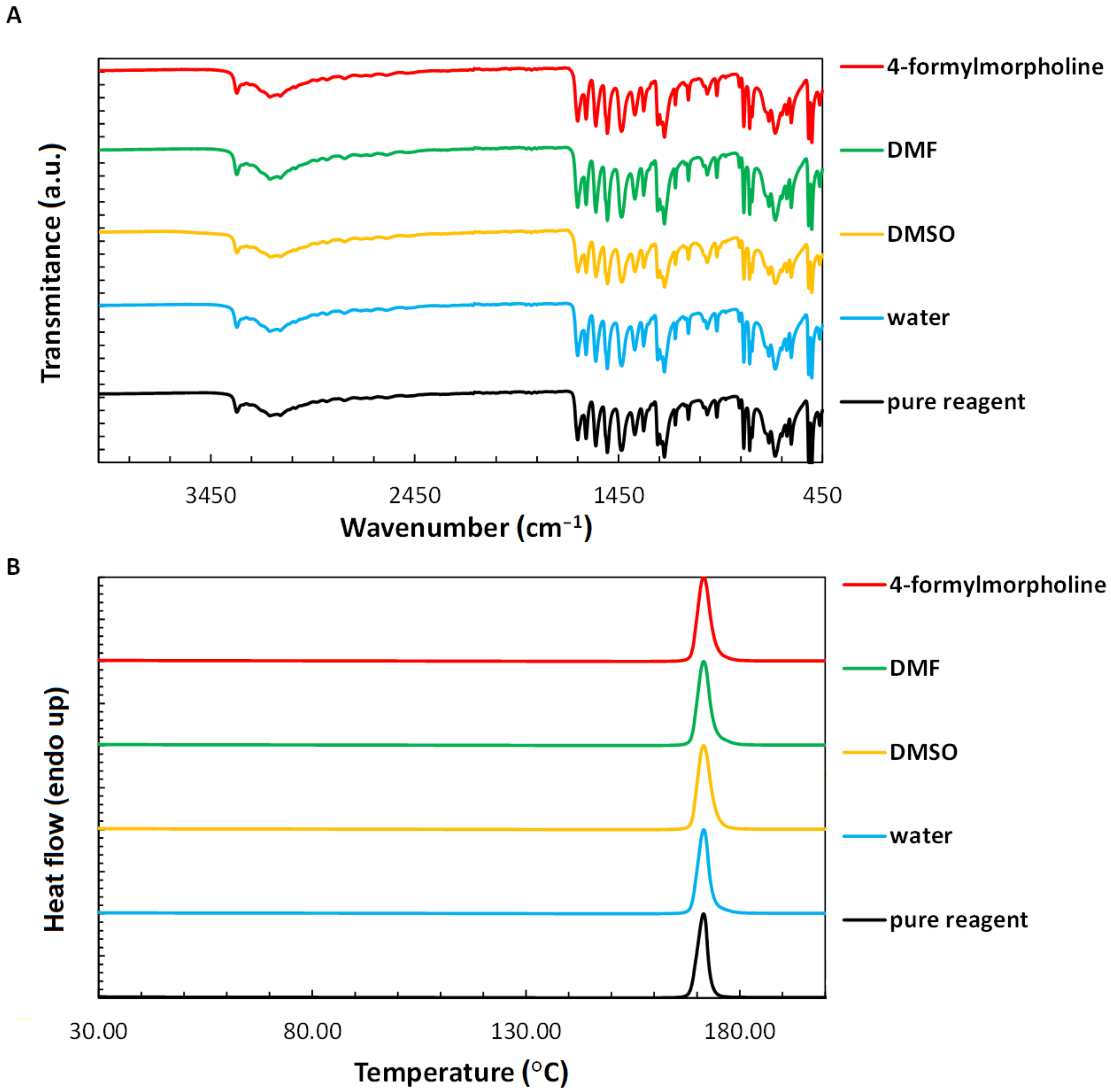


**Figure S1.** The FTIR-ATR spectra (A) and DSC thermograms (B) measured for acetaminophen solid residues and pure reagent.

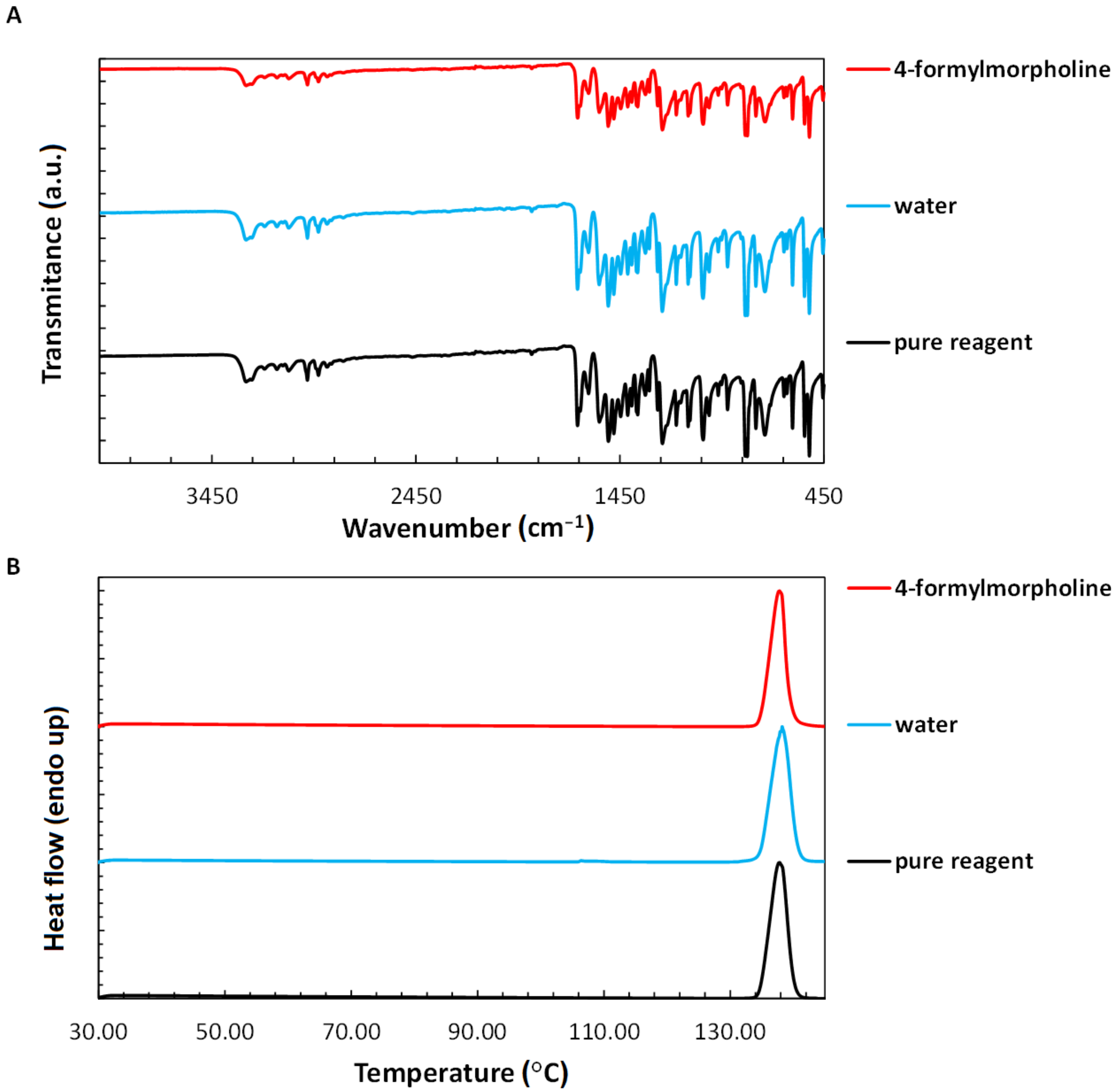


**Figure S2.** The FTIR-ATR spectra (A) and DSC thermograms (B) measured for phenacetin solid residues and pure reagent.

**Table S1.** The results of data curation for acetaminophen in neat solvents [1,2,11–20,3–10]. Data points were excluded from fitting λh-equation if PE>10%. The optimized values of parameters of the Buchowski-Ksiazczak [21] model applied to acetaminophen solubility in neat solvents are provided in the table in an additional supporting file (see Supplementary Materials S2 in "neat solvents").

| API+solvent | Solubility-temperature characteristics | API+solvent | Solubility-temperature characteristics |
|---|---|---|---|
| A+water [2,3,17–21,4–7,13–16] | 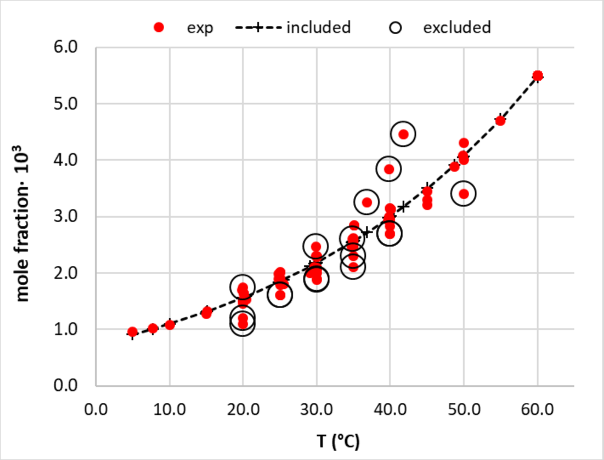 | A+methanol [6,8] | 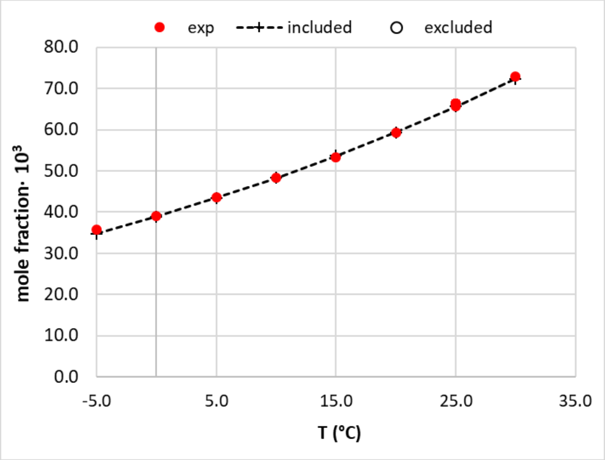 |
| A+ethanol [3,5,19] | 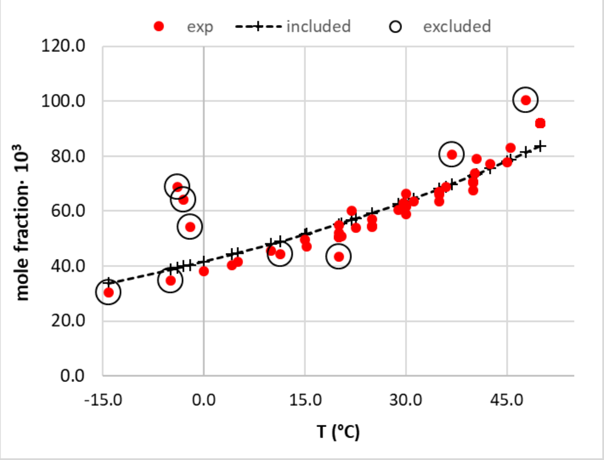 | A+1-propanol [8,14] | 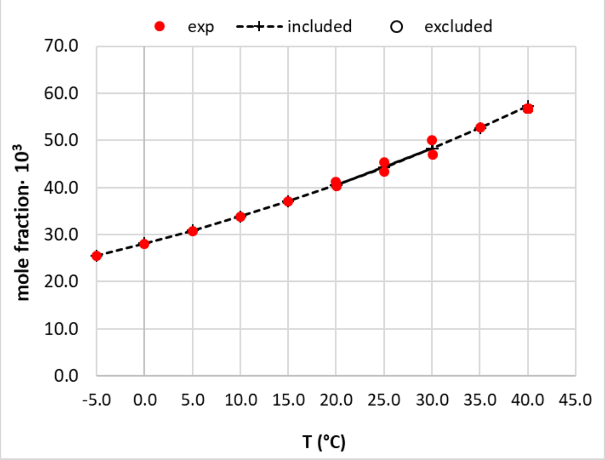 |

| API+solvent | Solubility-temperature characteristics | API+solvent | Solubility-temperature characteristics |
| --- | --- | --- | --- |
| A+2-propanol [8,9,15,16] | 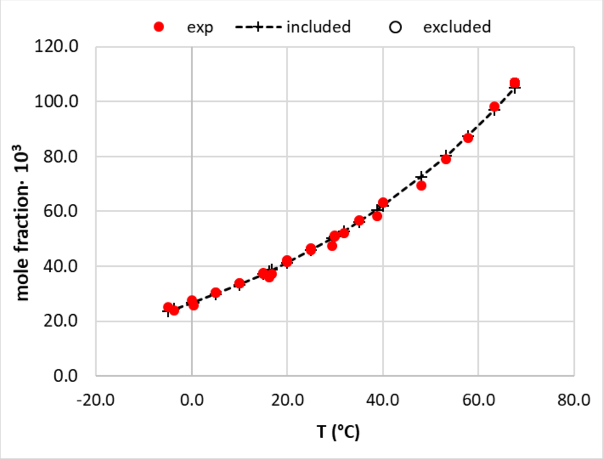 | A+1-butanol [8] | 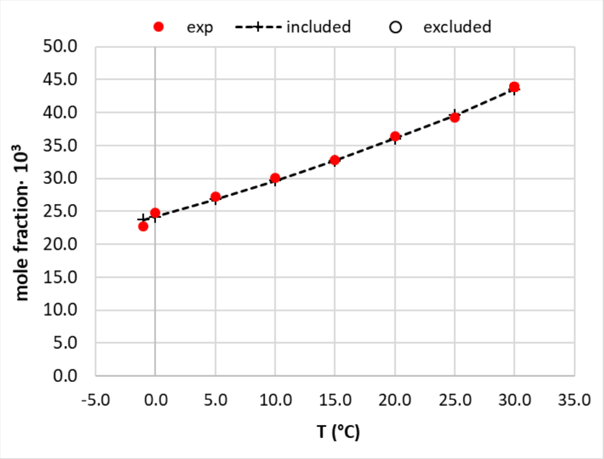 |
| A+1-octanol [9,10] | 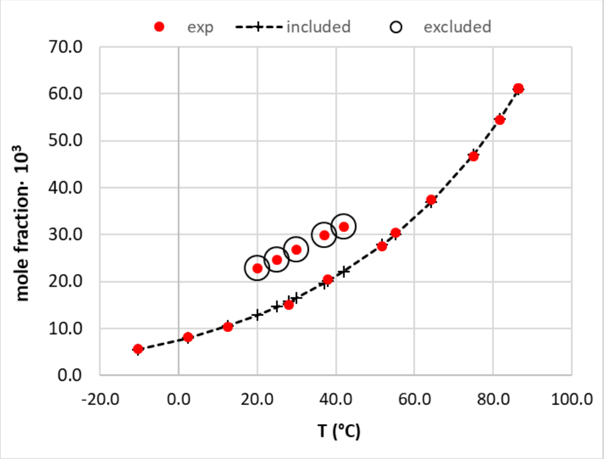 | A+propylene glycol [3–5] | 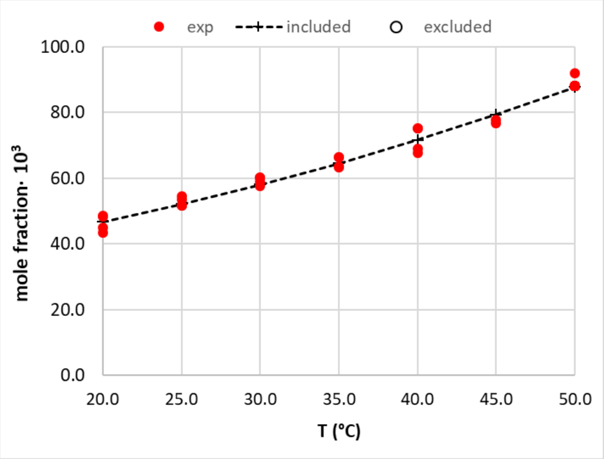 |

<table>
<tr><th>API+solvent</th><th>Solubility-temperature characteristics</th><th>API+solvent</th><th>Solubility-temperature characteristics</th></tr>
<tr><td>A+transcutol [5]</td><td>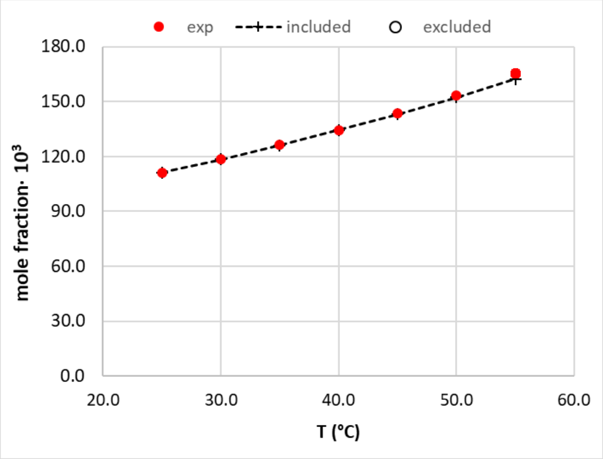
</td><td>A+ethyl acetate [8,11,19]</td><td>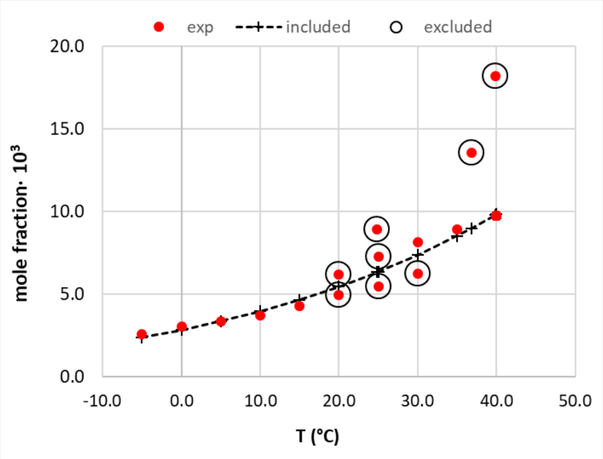
</td></tr>
<tr><td>A+isopropyl myristate [12]</td><td>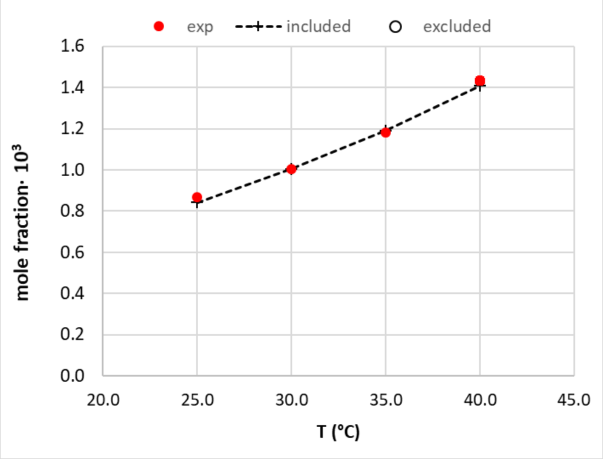
</td><td>A+acetone [8,11,16]</td><td>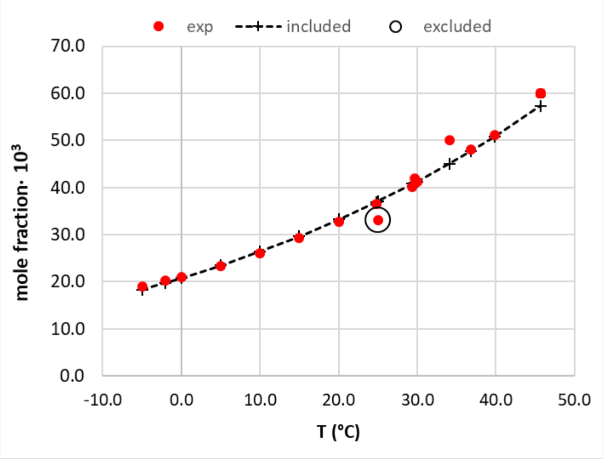
</td></tr>
</table>

<table>
<tr><th>API+solvent</th><th>Solubility-temperature characteristics</th><th>API+solvent</th><th>Solubility-temperature characteristics</th></tr>
<tr>
<td>A+acetonitrile [2,8]</td>
<td>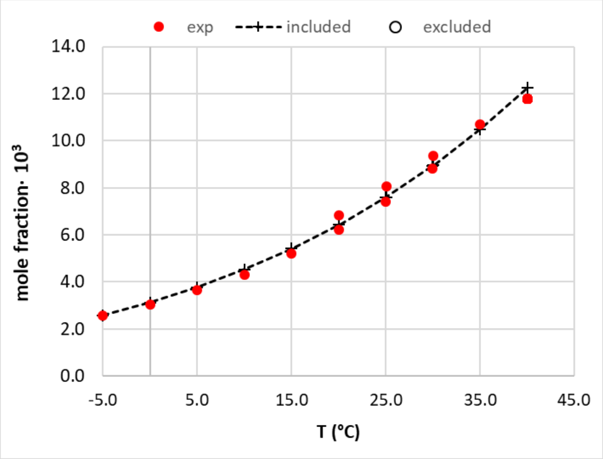
</td>
<td>A+1,4-dioxane [18]</td>
<td>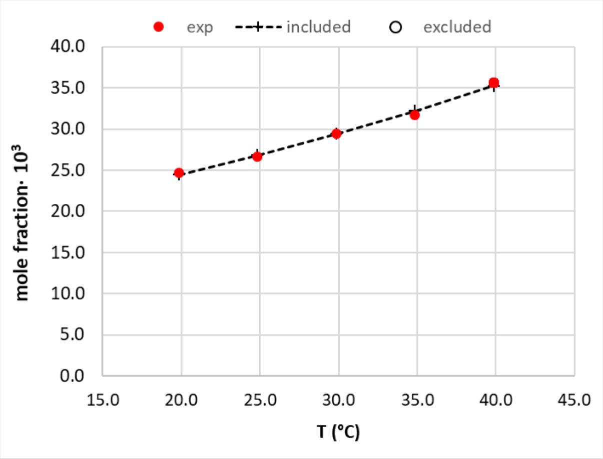
</td>
</tr>
<tr>
<td>A+hexane [10]</td>
<td>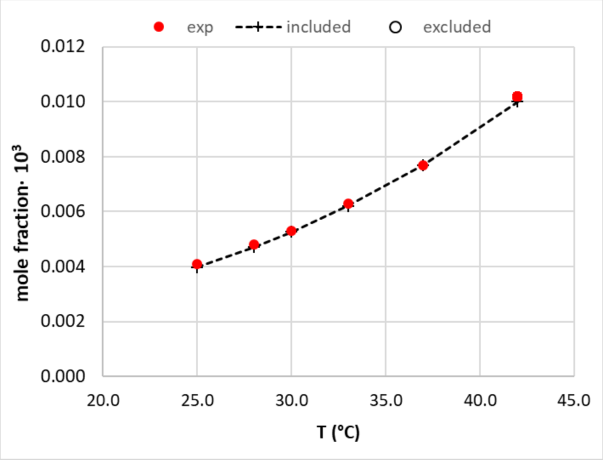
</td>
<td>A+cyclohexane [12]</td>
<td>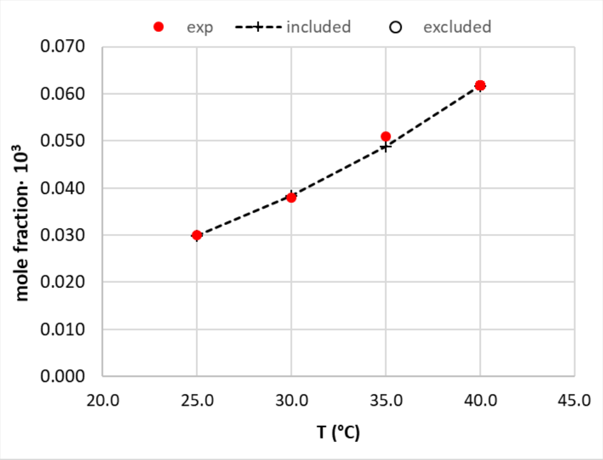
</td>
</tr>
</table>

<table>
<tr><th>API+solvent</th><th>Solubility-temperature characteristics</th><th>API+solvent</th><th>Solubility-temperature characteristics</th></tr>
<tr><td>A+chloroform [12]</td><td>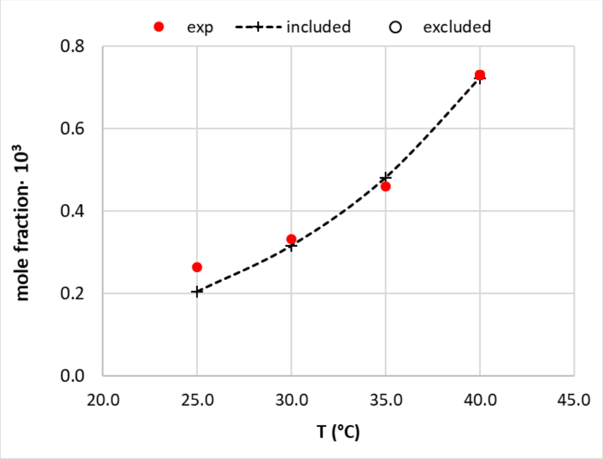
</td><td>A+DMSO [this work]</td><td>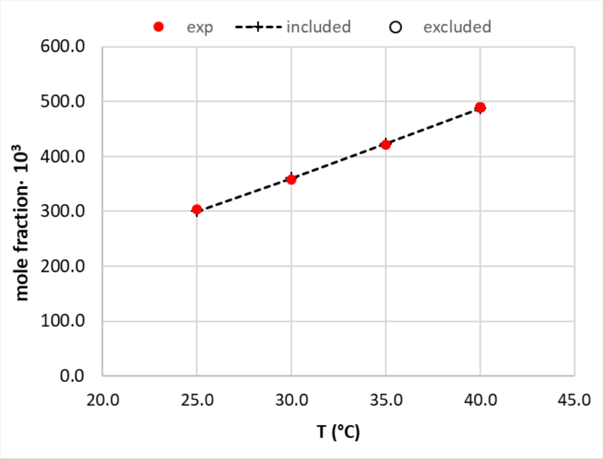
</td></tr>
<tr><td>A+4-formylmorpholine [this work]</td><td>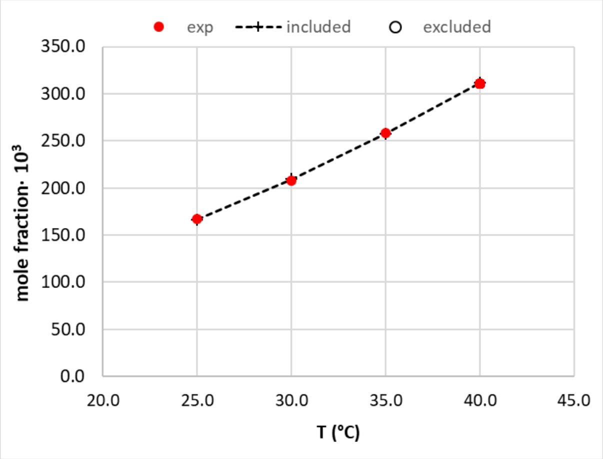
</td><td>A+DMF [this work]</td><td>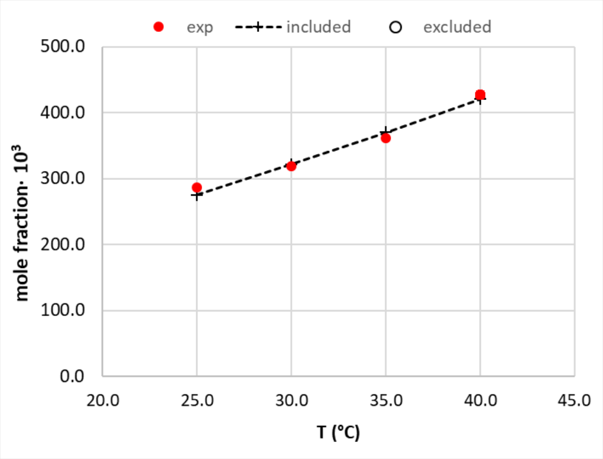
</td></tr>
</table>

**Table S2.** The results of data curation for phenacetin in neat solvents [19,22–25]. Data points were excluded from fitting λh-equation if PE>10%. The optimized values of parameters of the Buchowski-Ksiazczak [21] model applied to phenacetin solubility in neat solvents are provided in the table in an additional supporting file (see Supplementary Materials S2 in “neat solvents”).

<table>
<tr><th>API+solvent</th><th>Solubility-temperature characteristics</th><th>API+solvent</th><th>Solubility-temperature characteristics</th></tr>
<tr><td>P+water [12,22,23]</td><td>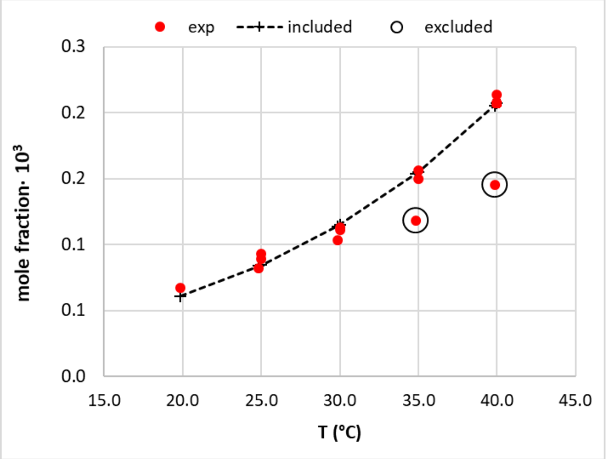
</td><td>P+methanol [23–25]</td><td>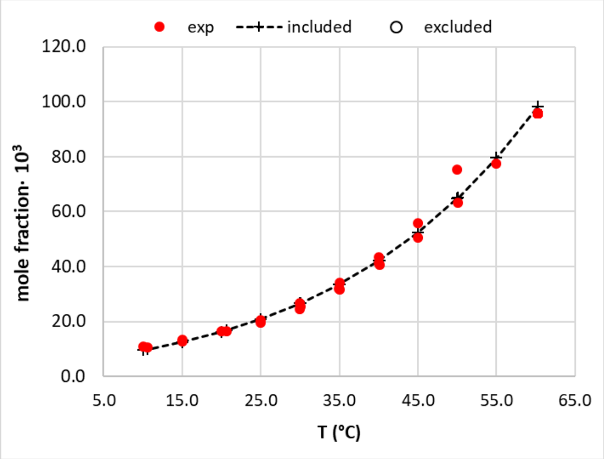
</td></tr>
<tr><td>P+ethanol [24,25]</td><td>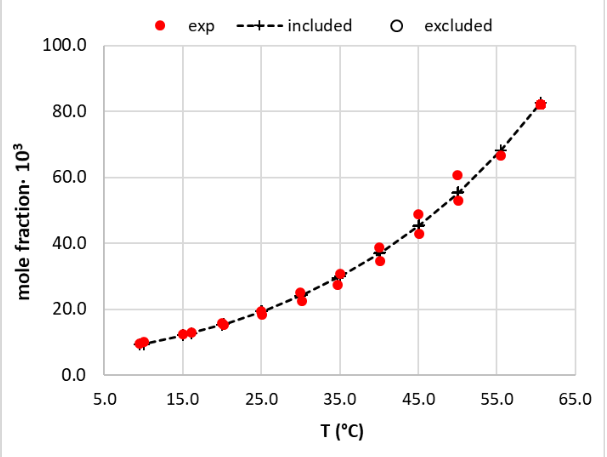
</td><td>P+1-propanol [24,25]</td><td>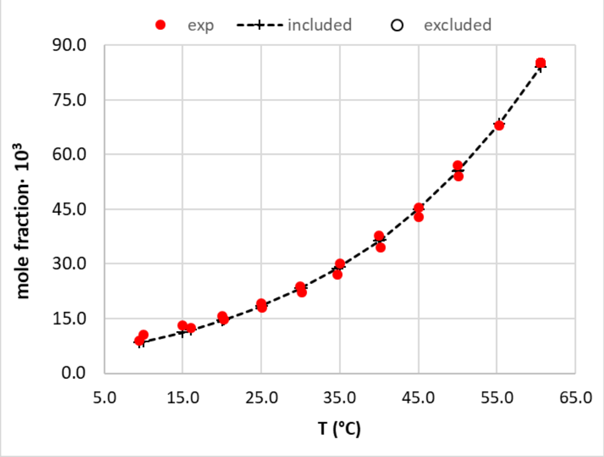
</td></tr>
</table>

| API+solvent | Solubility-temperature characteristics | API+solvent | Solubility-temperature characteristics |
|---|---|---|---|
| P+2-propanol [24] | 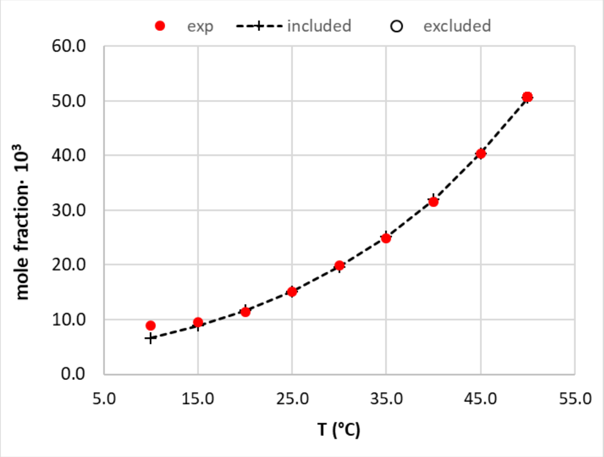 | P+1-butanol [24,25] | 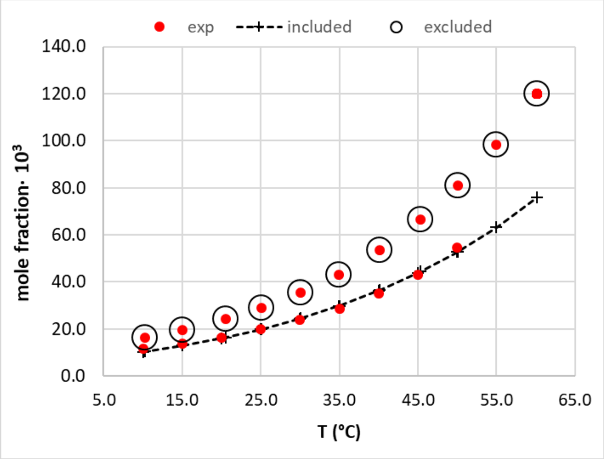 |
| P+2-butanol [24] | 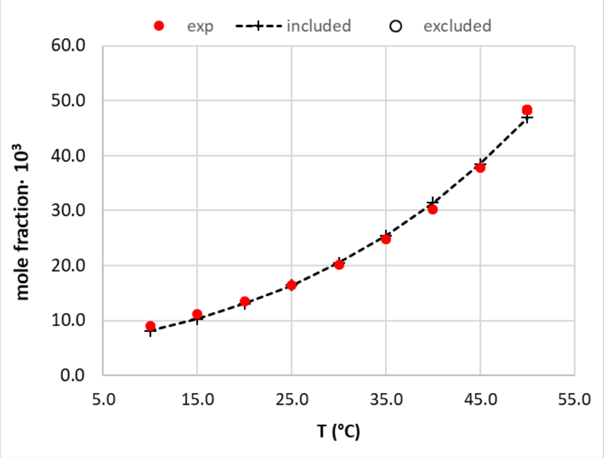 | P+1-pentanol [25] | 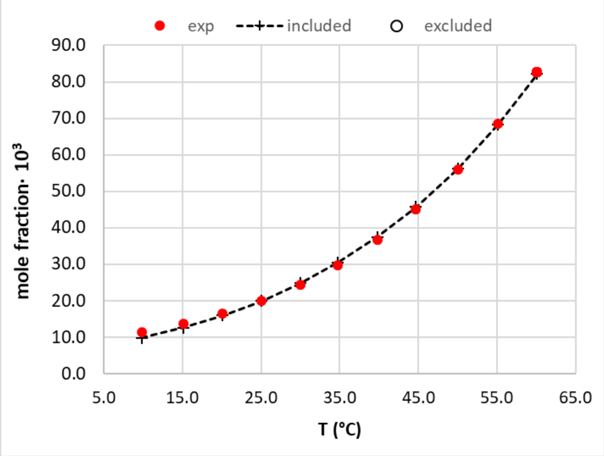 |

<table>
<tr><th>API+solvent</th><th>Solubility-temperature characteristics</th><th>API+solvent</th><th>Solubility-temperature characteristics</th></tr>
<tr><td>P+1-octanol [10]</td><td>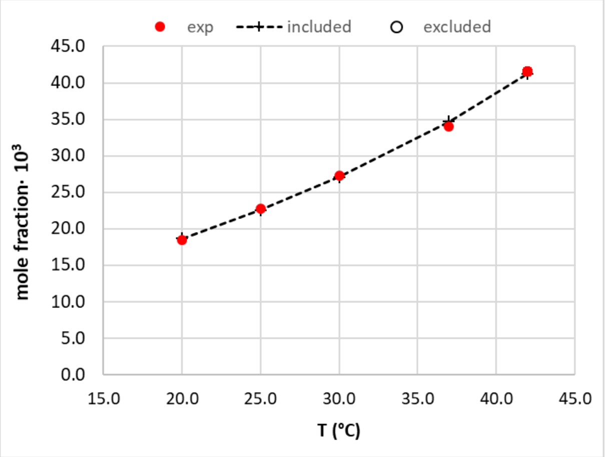
</td><td>P+ethyl formate [24]</td><td>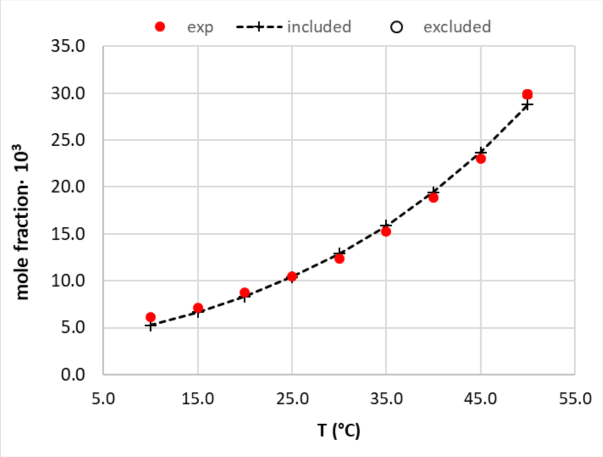
</td></tr>
<tr><td>P+ethyl acetate [24,25]</td><td>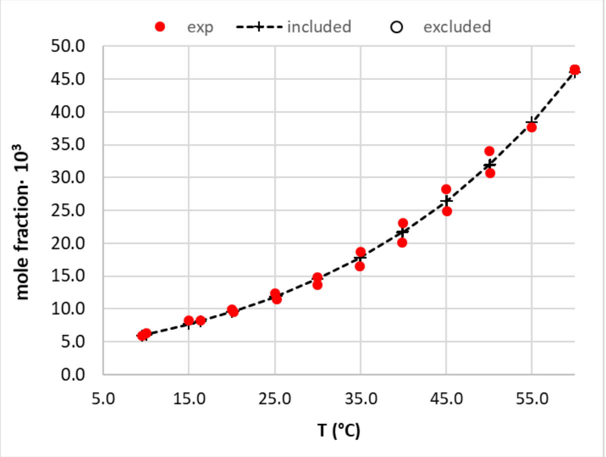
</td><td>P+n-propyl acetate [24]</td><td>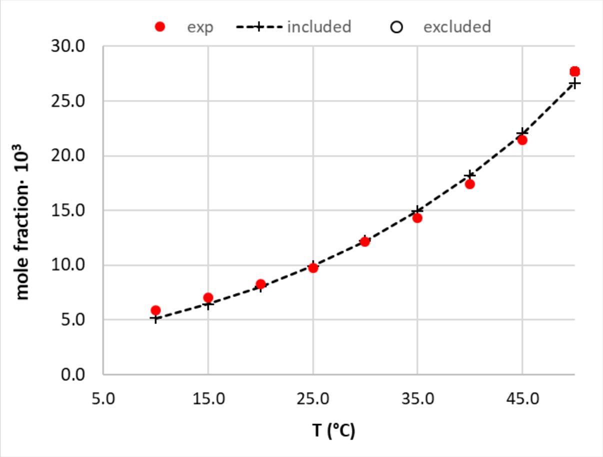
</td></tr>
</table>

| API+solvent | Solubility-temperature characteristics | API+solvent | Solubility-temperature characteristics |
|---|---|---|---|
| P+isopropyl myristate [12] | 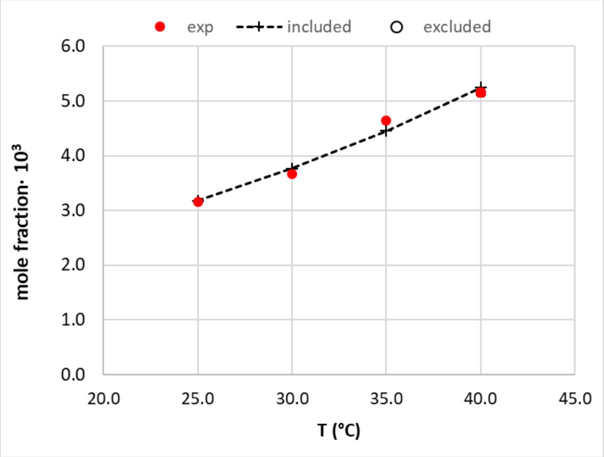 | P+acetonitrile [23,24] | 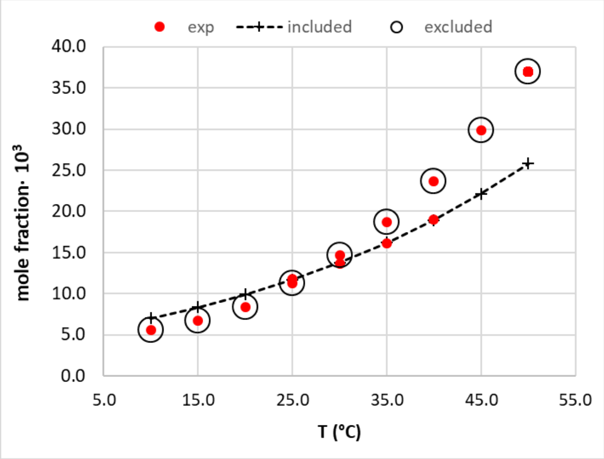 |
| P+1,4-dioxane [22,23] | 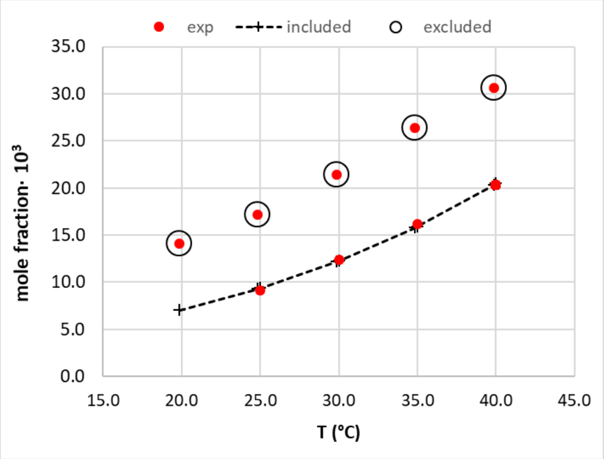 | P+4-formylmorpholine [this work] | 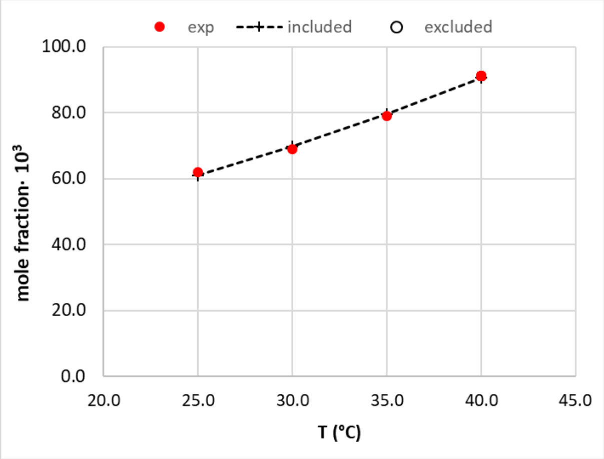 |

<table>
<tr><th>API+solvent</th><th>Solubility-temperature characteristics</th><th>API+solvent</th><th>Solubility-temperature characteristics</th></tr>
<tr><td>P+DMF [23,24]</td><td>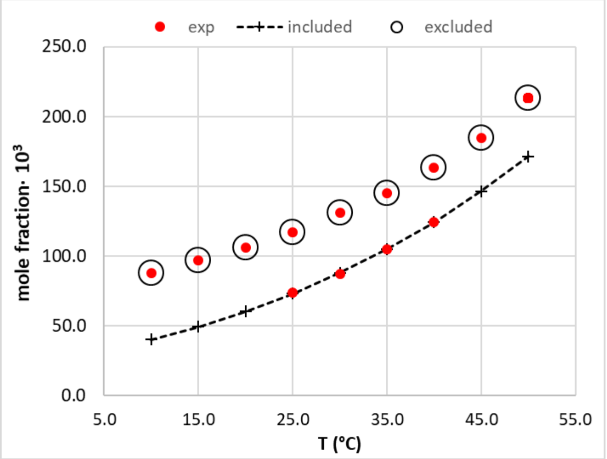
</td><td>P+DMSO [23]</td><td>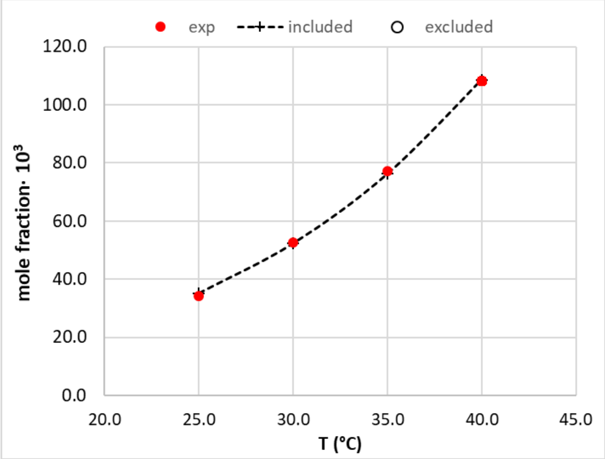
</td></tr>
<tr><td>P+N,N-dimethylacetamide [24]</td><td>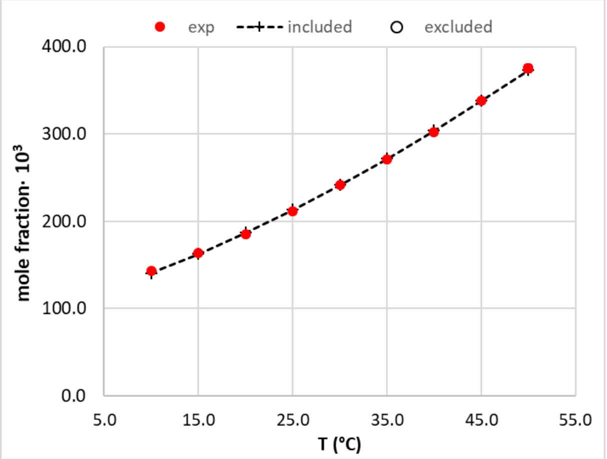
</td><td>P+THF [25]</td><td>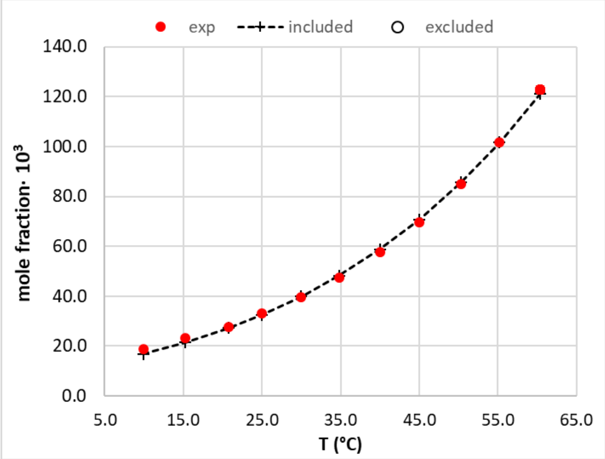
</td></tr>
</table>

<table>
<tr><th>API+solvent</th><th>Solubility-temperature characteristics</th><th>API+solvent</th><th>Solubility-temperature characteristics</th></tr>
<tr><td>P+hexane [10]</td><td>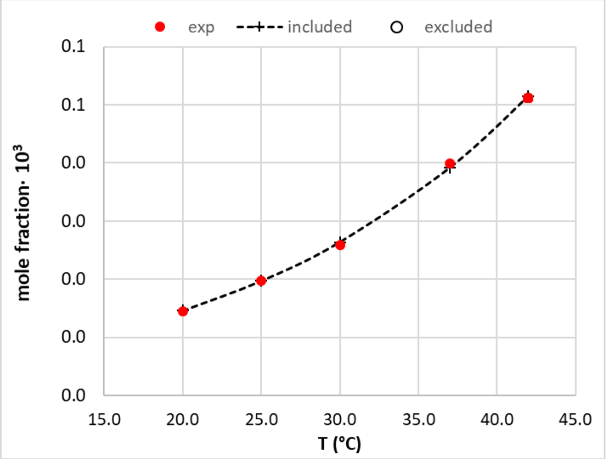
</td><td>P+cyclohexane [12]</td><td>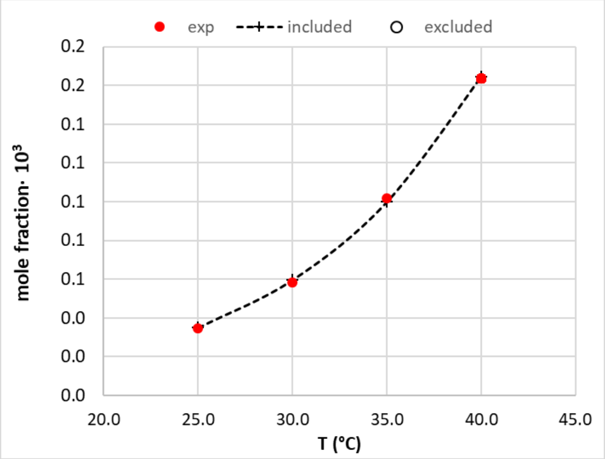
</td></tr>
<tr><td>P+benzene [25]</td><td>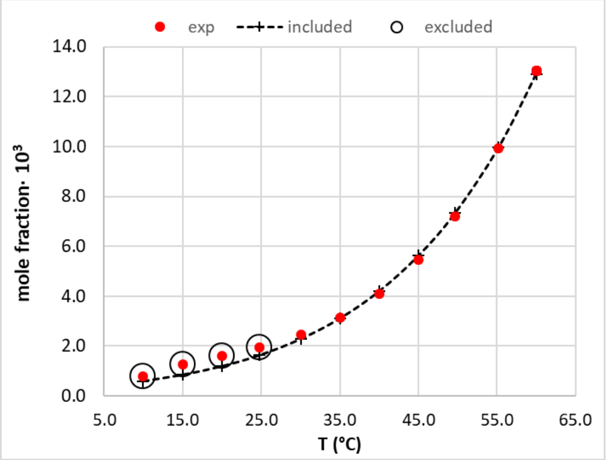
</td><td>P+chloroform [12]</td><td>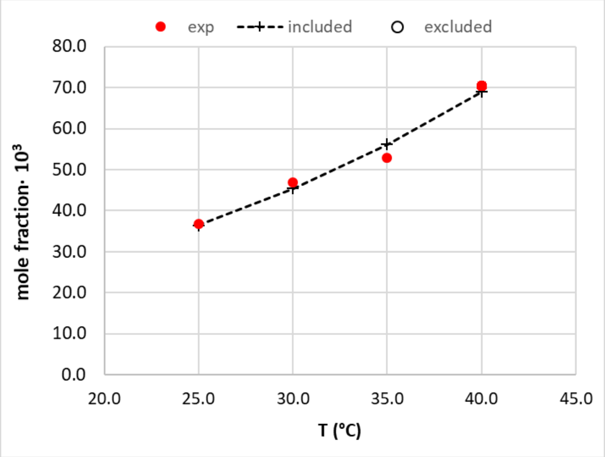
</td></tr>
</table>

**Table S3.** The data collection of acetaminophen solubility in binary solvent mixtures [1,2,13,14,26,3,4,7–12]. The optimized values of parameters of the Jouyban-Acree model [27] model applied to acetaminophen solubility in binary solvents are provided in the table in an additional supporting file (see Supplementary Materials S2 in "binary solvents").

| API+solvent | Solubility vs. binary solvent composition | API+solvent | Solubility vs. binary solvent composition |
|---|---|---|---|
| A+methanol+water [6] | 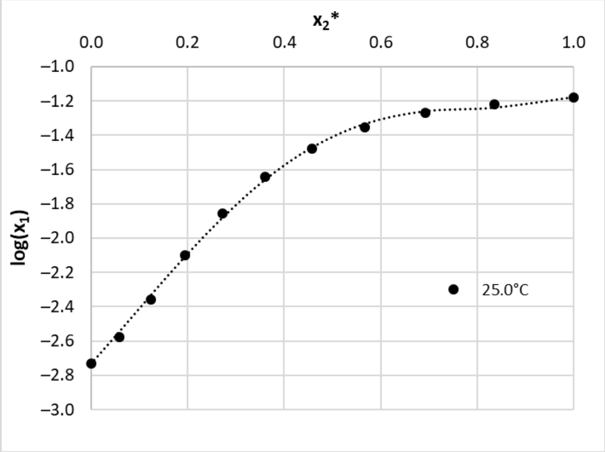  | A+ethanol+water [3,5,19,21,26] | 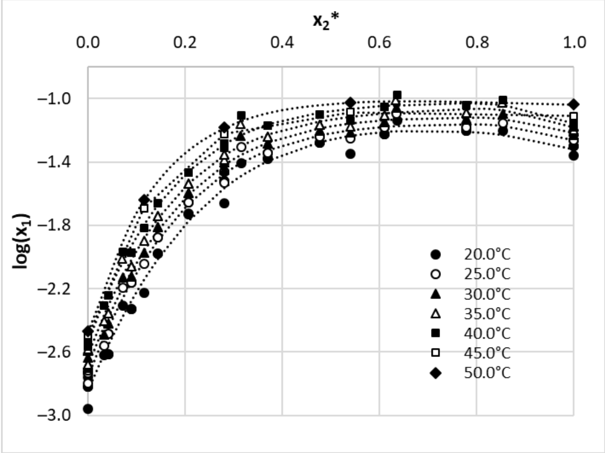  |
| A+ acetaminophen+propanol [14] (*) | 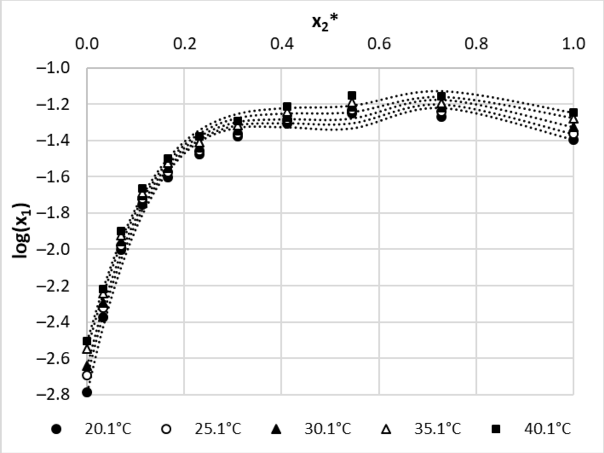  | A+ isopropanol+water [15] (*) | 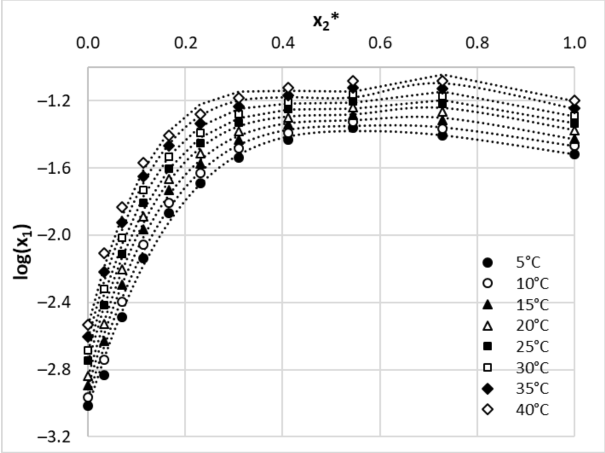  |

<table>
<tr><th>API+solvent</th><th>Solubility vs. binary solvent composition</th><th>API+solvent</th><th>Solubility vs. binary solvent composition</th></tr>
<tr><td>A+propylene glycol+water [4][3][5]</td><td>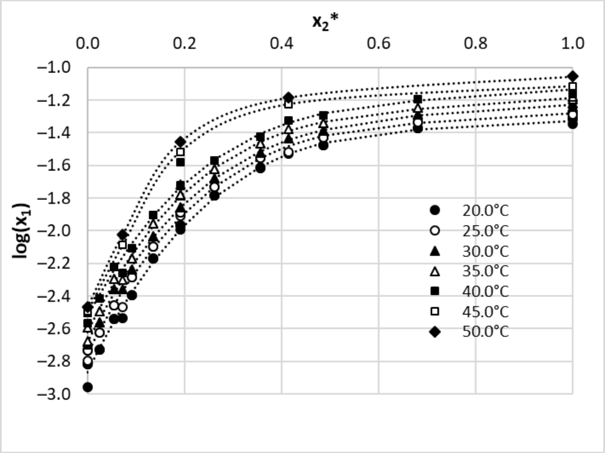
</td><td>A+transcutol+water [7](*)</td><td>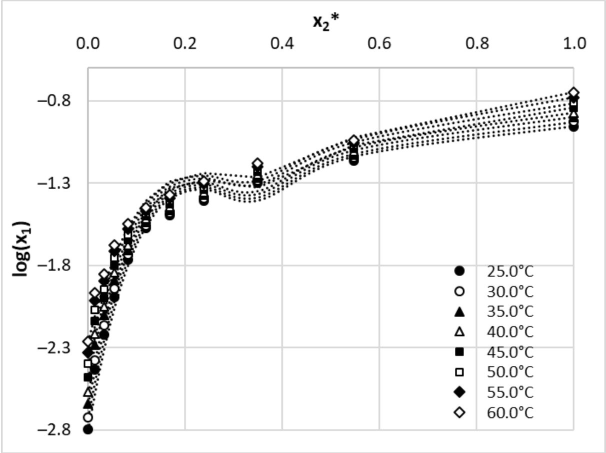
</td></tr>
<tr><td>A+acetonitrile+water [2]</td><td>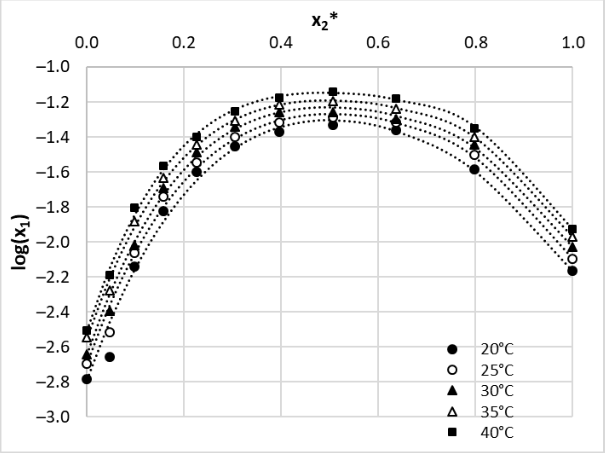
</td><td>A+1,4-dioxane+water [18] (*)</td><td>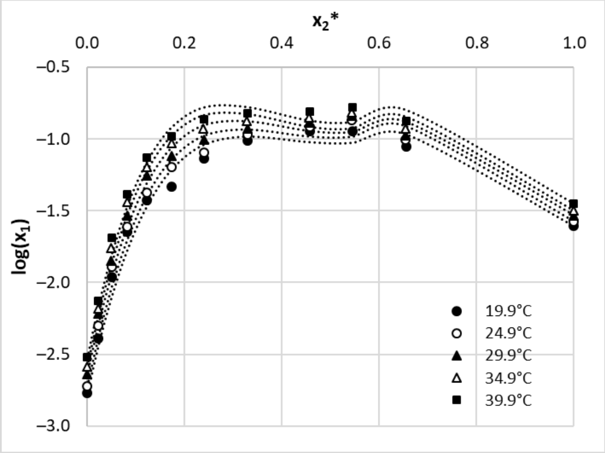
</td></tr>
</table>

<table>
<tr><th>API+solvent</th><th>Solubility vs. binary solvent composition</th><th>API+solvent</th><th>Solubility vs. binary solvent composition</th></tr>
<tr><td>A+ethanol+propylene glycol [20]</td><td>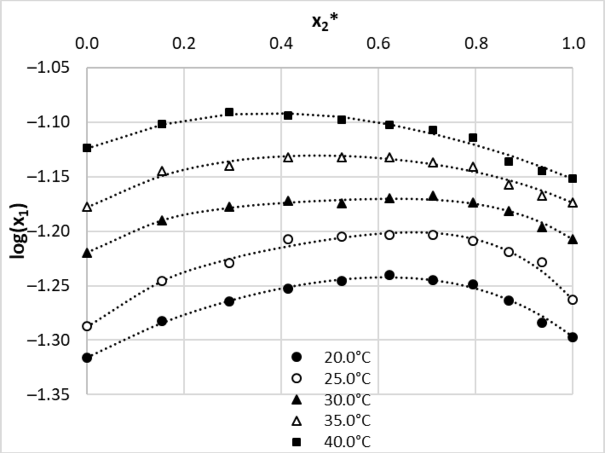
</td><td>A+ethanol+ethyl acetate [19]</td><td>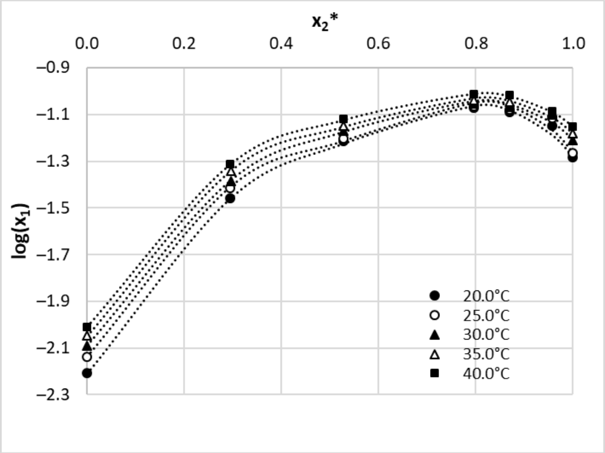
</td></tr>
<tr><td>A+4-formylmorpholine +water [this work]</td><td>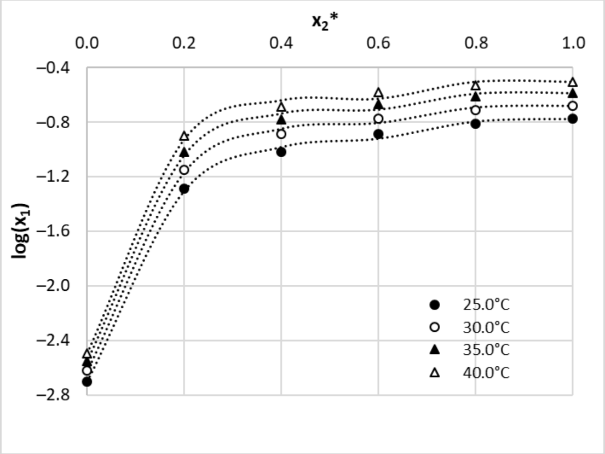
</td><td>A+DMF+water [this work]</td><td>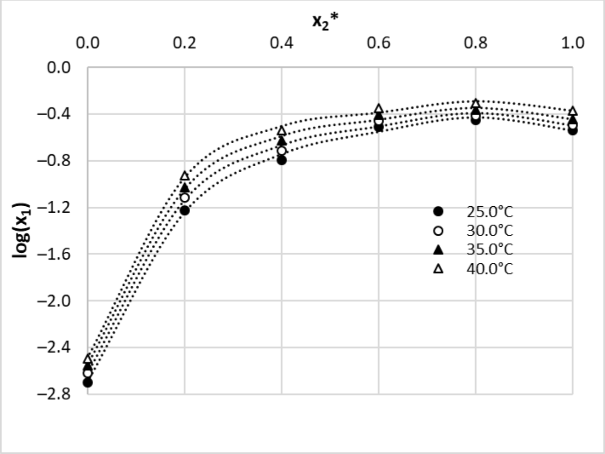
</td></tr>
</table>

| API+solvent | Solubility vs. binary solvent composition | API+solvent | Solubility vs. binary solvent composition |
|---|---|---|---|
| A+DMSO+water [this work] | 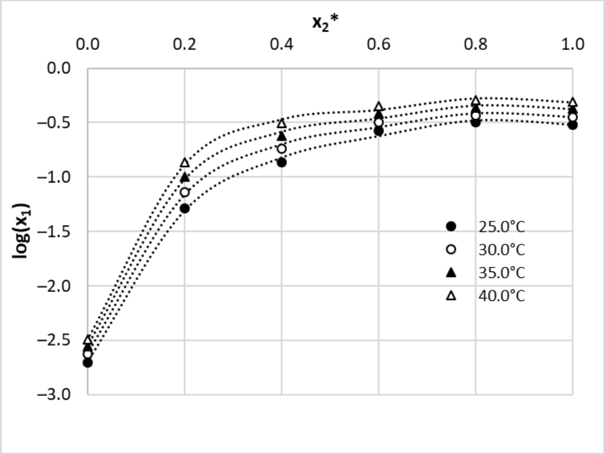 | | |

(*)these system challenged the JA model leading to inaccurate back-computed solubility values in regions of high concentration of organic solvent.

**Table S4.** The data collection of phenacetin solubility in binary solvent mixtures [22,23,28]. The optimized values of parameters of the Jouyban-Acree model [27] model applied to phenacetin solubility in binary solvents are provided in the table in an additional supporting file (see Supplementary Materials S2 in "binary solvents").

<table>
<tr><th>API+solvent</th><th>Solubility vs. binary solvent composition</th><th>API+solvent</th><th>Solubility vs. binary solvent composition</th></tr>
<tr><td>P+methanol+water [23,27]</td><td>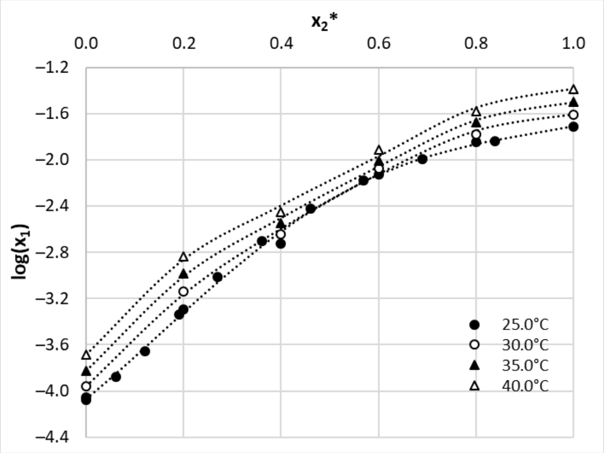
</td><td>P+1,4-dioxane+water [22,23] (*)</td><td>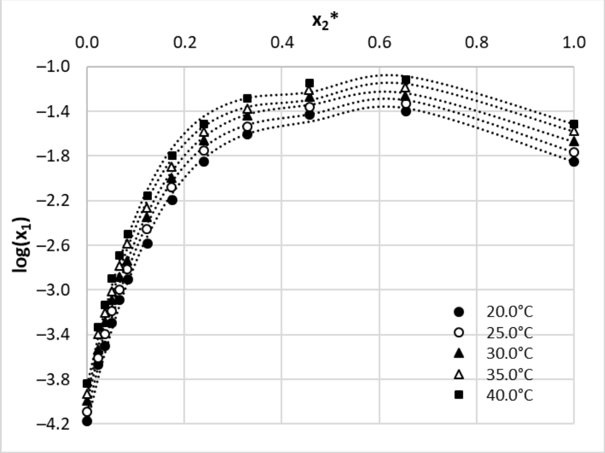
</td></tr>
<tr><td>P+acetonitrile+water [23]</td><td>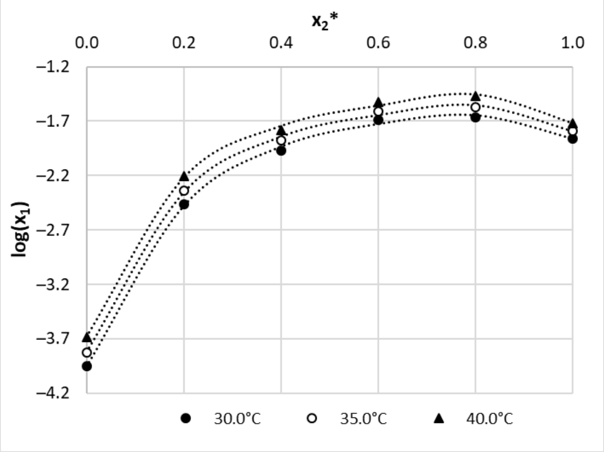
</td><td>P+DMF+water [23] (*)</td><td>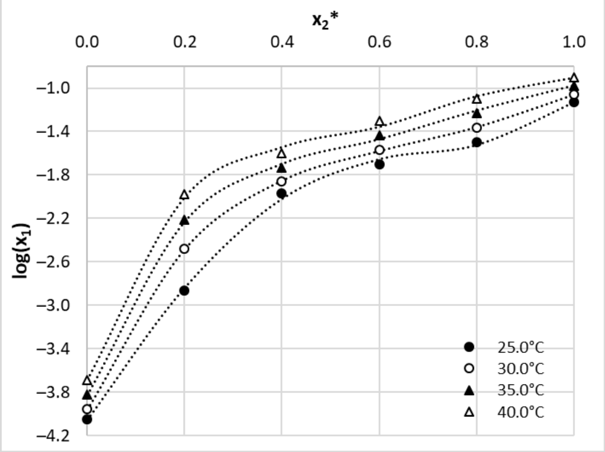
</td></tr>
</table>

<table>
<tr><th>API+solvent</th><th>Solubility vs. binary solvent composition</th><th>API+solvent</th><th>Solubility vs. binary solvent composition</th></tr>
<tr><td>P+DMSO+water [23]</td><td>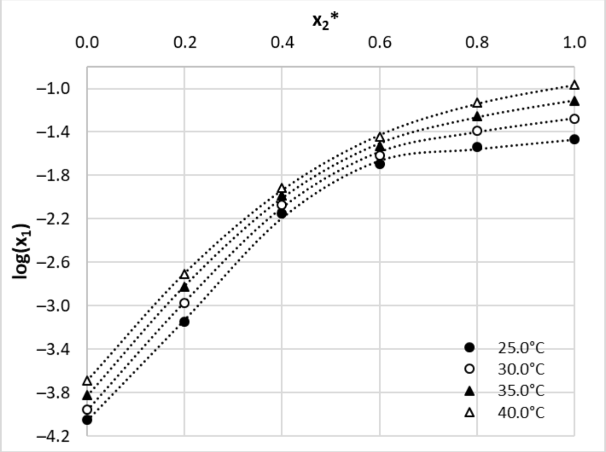
</td><td>P+4-formylmorpholine+ water [this work]</td><td>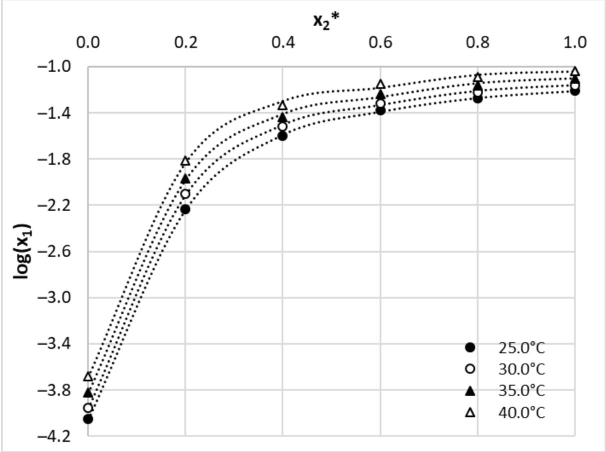
</td></tr>
</table>

(*)these system challenged the JA model leading to inaccurate back-computed solubility values in regions of high concentration of organic solvent.

## References


1. Rahimpour, E.; Mazaher Haji Agha, E.; Martinez, F.; Barzegar-Jalali, M.; Jouyban, A. Solubility study of acetaminophen in the mixtures of acetonitrile and water at different temperatures. *J. Mol. Liq.* **2021**, *324*, 114708.
2. Romdhani, A.; Martínez, F.; Almanza, O.A.; Jouyban, A.; Acree, W.E. Solubility of acetaminophen in (ethanol + propylene glycol + water) mixtures: Measurement, correlation, thermodynamics, and volumetric contribution at saturation. *J. Mol. Liq.* **2020**, *318*, 114065.
3. Pourkarim, F.; Mirheydari, S.N.; Martinez, F.; Jouyban, A. Solubility of acetaminophen in 1-propanol + water mixtures at T = 293.2–313.2 K. *Phys. Chem. Liq.* **2020**, *58*, 456–472.
4. Hojjati, H.; Rohani, S. Measurement and prediction of solubility of paracetamol in water-isopropanol solution. Part 1. Measurement and data analysis. *Org. Process Res. Dev.* **2006**, *10*, 1101–1109.
5. Hahnenkamp, I.; Graubner, G.; Gmehling, J. Measurement and prediction of solubilities of active pharmaceutical ingredients. *Int. J. Pharm.* **2010**, *388*, 73–81.
6. Pascual, G.K.; Donnellan, P.; Glennon, B.; Kamaraju, V.K.; Jones, R.C. Experimental and Modeling Studies on the Solubility of 2-Chloro-N-(4-methylphenyl)propanamide (S1) in Binary Ethyl Acetate + Hexane, Toluene + Hexane, Acetone + Hexane, and Butanone + Hexane Solvent Mixtures Using Polythermal Method. *J. Chem. Eng. Data* **2017**, *62*, 3193–3205.
7. Bustamante, P. Enthalpy–entropy compensation for the solubility of drugs in solvent mixtures: Paracetamol, acetanilide, and nalidixic acid in dioxane–water. *J. Pharm. Sci.* **1998**, *87*, 1590–1596.
8. Bustamante, P.; Romero, S.; Reillo, A. Thermodynamics of Paracetamol in Amphiprotic and Amphiprotic—aprotic Solvent Mixtures. *Pharm. Pharmacol. Commun.* **1995**, *1*, 505–507.
9. Jiménez, J.A.; Martínez, F. Temperature Dependence of the Solubility of Acetaminophen in Propylene Glycol + Ethanol Mixtures. *J. Solut. Chem.* **2006**, *35*, 335–352.
10. Jiménez, J.A.; Martínez, F. Thermodynamic magnitudes of mixing and solvation of acetaminophen in ethanol + water cosolvent mixtures. *Rev. acad. colomb. cienc.* **2006**, *30*, 87–99.
11. Jiménez, J.A.; Martínez, F. Thermodynamic study of the solubility of acetaminophen in propylene glycol + water cosolvent mixtures. *J. Braz. Chem. Soc.* **2006**, *17*, 125–134.
12. Assis, G.P.; Garcia, R.H.L.; Derenzo, S.; Bernardo, A. Solid-liquid equilibrium of paracetamol in water-ethanol and water-propylene glycol mixtures. *J. Mol. Liq.* **2021**, *323*, 114617.
13. Muñoz, M.M.; Jouyban, A.; Martínez, F. Solubility and preferential solvation of acetaminophen in methanol + water mixtures at 298.15 K. *Phys. Chem. Liq.* **2015**, *54*, 515–528.
14. Shakeel, F.; Alanazi, F.K.; Alsarra, I.A.; Haq, N. Solubilization behavior of paracetamol in transcutol-water mixtures at (298.15 to 333.15) K. *J. Chem. Eng. Data* **2013**, *58*, 3551–3556.
15. Granberg, R.A.; Rasmuson, Å.C. Solubility of paracetamol in pure solvents. *J. Chem. Eng. Data* **1999**, *44*, 1391–1395.
16. Matsuda, H.; Mori, K.; Tomioka, M.; Kariyasu, N.; Fukami, T.; Kurihara, K.; Tochigi, K.; Tomono, K. Determination and prediction of solubilities of active pharmaceutical ingredients in selected organic solvents. *Fluid Phase Equilib.* **2015**, *406*, 116–123.
17. Perlovich, G.L.; Volkova, T. V.; Bauer-Brandl, A. Towards an understanding of the molecular mechanism of solvation of drug molecules: A thermodynamic approach by crystal lattice energy, sublimation, and solubility exemplified by paracetamol, acetanilide, and phenacetin. *J. Pharm. Sci.* **2006**, *95*, 2158–2169.
18. Mota, F.L.; Carneiro, A.P.; Queimada, A.J.; Pinho, S.P.; Macedo, E.A. Temperature and solvent effects in the solubility of some pharmaceutical compounds: Measurements and modeling. *Eur. J. Pharm. Sci.* **2009**, *37*, 499–507.
19. Baena, Y.; Pinzón, J.A.; Barbosa, H.J.; Martínez, F. Temperature-dependence of the solubility of some acetanilide derivatives in several organic and aqueous solvents. *Phys. Chem. Liq.* **2004**, *42*, 603–613.
20. Hajebrahimi, S.; Roosta, A. Solubility of acetaminophen in aqueous solutions of three natural deep eutectic solvents (NADESs) and individual components of the NADESs. *J. Mol. Liq.* **2020**, *316*, 113867.
21. Buchowski, H.; Ksiazczak, A.; Pietrzyk, S. Solvent activity along a saturation line and solubility of

hydrogen-bonding solids. *J. Phys. Chem.* **1980**, *84*, 975–979.
22. Bustamante, C.; Bustamante, P. Nonlinear Enthalpy–Entropy Compensation for the Solubility of Phenacetin in Dioxane–Water Solvent Mixtures. *J. Pharm. Sci.* **1996**, *85*, 1109–1111.
23. Przybyłek, M.; Kowalska, A.; Tymorek, N.; Dziaman, T.; Cysewski, P. Thermodynamic characteristics of phenacetin in solid state and saturated solutions in several neat and binary solvents. *Molecules* **2021**, *26*, 4078.
24. Wang, L.; Li, D.; Wang, L.; Hao, H.; Zhou, L. Measurement and Correlation of Solubility and Thermodynamic Properties of Phenacetin in 12 Pure Solvents from 283.15 to 323.15 K. *J. Chem. Eng. Data* **2021**, *66*, 4593–4602.
25. Chang, Q.L.; Li, Q.S.; Wang, S.; Tian, Y.M. Solubility of phenacetinum in methanol, ethanol, 1-propanol, 1-butanol, 1-pentanol, tetrahydrofuran, ethyl acetate, and benzene between 282.65 K and 333.70 K. *J. Chem. Eng. Data* **2007**, *52*, 1894–1896.
26. Prakongpan, S.; Nagai, T. Solubility of Acetaminophen in Cosolvents. *Chem. Pharm. Bull.* **1984**, *32*, 340–343.
27. Jouyban, A.; Acree, W.E. Mathematical derivation of the Jouyban-Acree model to represent solute solubility data in mixed solvents at various temperatures. *J. Mol. Liq.* **2018**, *256*, 541–547.
28. Cárdenas, Z.J.; Almanza, O.A.; Jouyban, A.; Martínez, F.; Acree, W.E. Solubility and preferential solvation of phenacetin in methanol + water mixtures at 298.15 K. *Phys. Chem. Liq.* **2018**, *56*, 16–32.
